# Translational derepression of *Elavl4* isoforms at their alternative 5′ UTRs determines neuronal development

**DOI:** 10.1038/s41467-020-15412-8

**Published:** 2020-04-03

**Authors:** Tatiana Popovitchenko, Yongkyu Park, Nicholas F. Page, Xiaobing Luo, Zeljka Krsnik, Yuan Liu, Iva Salamon, Jessica D. Stephenson, Matthew L. Kraushar, Nicole L. Volk, Sejal M. Patel, H. R. Sagara Wijeratne, Diana Li, Kandarp S. Suthar, Aaron Wach, Miao Sun, Sebastian J. Arnold, Wado Akamatsu, Hideyuki Okano, Luc Paillard, Huaye Zhang, Steven Buyske, Ivica Kostovic, Silvia De Rubeis, Ronald P. Hart, Mladen-Roko Rasin

**Affiliations:** 1 Department of Neuroscience and Cell Biology, Rutgers University, Robert Wood Johnson Medical School, Piscataway, NJ 08854 USA; 2 Graduate Program in Neurosciences, Rutgers University, Piscataway, NJ 08854 USA; 3 Department of Cell Biology and Neuroscience, Rutgers University, Piscataway, NJ 08854 USA; 4 Croatian Institute for Brain Research, Center of Research Excellence for Basic, Clinical and Translational Neuroscience, University of Zagreb, School of Medicine, Zagreb, 10000 Croatia; 5 Institute of Experimental and Clinical Pharmacology and Toxicology, Faculty of Medicine, Signaling Research Centers BIOSS and CIBSS, University of Freiburg, Freiburg, D-79104 Germany; 6 Department of Physiology, Keio University School of Medicine, Tokyo, 160-8582 Japan; 7 Univ Rennes, CNRS, IGDR (Institut de génétique et développement de Rennes)-UMR 6290, F-35000 Rennes, France; 8 Department of Statistics, Rutgers University, Piscataway, NJ 08854 USA; 9 Department of Psychiatry, Icahn School of Medicine at Mount Sinai, 1 Gustave L. Levy Pl, New York, NY 10029 USA; 10 Seaver Autism Center, Icahn School of Medicine at Mount Sinai, New York, NY 10029 USA; 11 Mindich Child Health and Development Institute, Icahn School of Medicine at Mount Sinai, New York, NY 10029 USA; 12 Friedman Brain Institute, Icahn School of Medicine at Mount Sinai, New York, NY 10029 USA

**Keywords:** Cell signalling, Development of the nervous system

## Abstract

Neurodevelopment requires precise regulation of gene expression, including post-transcriptional regulatory events such as alternative splicing and mRNA translation. However, translational regulation of specific isoforms during neurodevelopment and the mechanisms behind it remain unknown. Using RNA-seq analysis of mouse neocortical polysomes, here we report translationally repressed and derepressed mRNA isoforms during neocortical neurogenesis whose orthologs include risk genes for neurodevelopmental disorders. We demonstrate that the translation of distinct mRNA isoforms of the RNA binding protein (RBP), *Elavl4*, in radial glia progenitors and early neurons depends on its alternative 5′ UTRs. Furthermore, 5′ UTR-driven *Elavl4* isoform-specific translation depends on upstream control by another RBP, Celf1. Celf1 regulation of *Elavl4* translation dictates development of glutamatergic neurons. Our findings reveal a dynamic interplay between distinct RBPs and alternative 5′ UTRs in neuronal development and underscore the risk of post-transcriptional dysregulation in co-occurring neurodevelopmental disorders.

## Abstract (web summary)

Translational regulation of isoforms in the developing nervous system is not well understood. Here, the authors report translational de-repression of RNA binding protein isoforms at their 5′UTRs in the neocortex and show the neurodevelopmental risk of post-transcriptional dysregulation.

## Introduction

The mature neocortex generates complex behaviors including high-level cognition and voluntary motor functions. In the prenatal neocortex, neural stem cells called radial glia progenitors (RG) sequentially give rise to distinct subpopulations of glutamatergic neurons that are critical for normal circuits and functions^1–3^. RG and glutamatergic neurons execute transcriptional and post-transcriptional programs that drive their development^1–7^. Post-transcriptional regulatory programs include alternative splicing of mRNA isoforms that determine cell fate^8^ and the regulation of mRNA translation (protein synthesis)^2,3,5,7,9–15^. These two mechanisms are interconnected, as alternative mRNA sequences may yield distinct interactions with translational machinery.

Translational control is facilitated by RNA-binding proteins (RBPs), which often act on regulatory elements found in 5′ and 3′ untranslated regions (UTRs)^3,5,7,16–18^. UTRs are sites where multiple trans-acting factors may compete or cooperate to affect translation^19,20^. Alternatively spliced 3′ UTRs not only influence translation efficiency, but also modify the localization and stability of mRNAs in neurons^18^. Alternative 5′ UTRs are upstream of the start codon and are in a powerful position to dictate translational efficiency of a given mRNA^17,21^. Yet, the effect of alternative 5′ UTRs on translation and how multiple RBPs interact with them to dictate neuronal development are unknown.

Two RBPs in the Embryonic Lethal, Abnormal Vision, Drosophila, homolog-Like 4 family, Elavl4 (HuD) and Elavl1 (HuR), regulate neocortical development and mRNA translation^9,10,13^. Elavl4 deletion in mice results in seizures and repetitive behaviors^9^ reminiscent of phenotypes observed in individuals with comorbid epileptic encephalopathy (EE) and autism spectrum disorder (ASD). These behavioral anomalies are accompanied by anatomically disrupted glutamatergic neurons^9^. Another RBP, the CUGBP Elavl Family Member 1 (Celf1/Cugbp1), is highly expressed during neocortical development^5,22^. While Celf1 mediates multiple post-transcriptional steps in other systems^23–25^, its role in developing neocortical progenitors and neurons remains unknown. In addition, it is still poorly understood if and how distinct mRNA isoforms are translationally regulated in space and time during neuronal development.

Here, we provide a genome-wide screen of mRNA isoforms during prenatal neocortical neurogenesis and their translational patterns of repression and de-repression. Remarkably, human orthologues of many of these translationally-regulated mRNAs are encoded by known risk genes for comorbid neurodevelopmental disorders (e.g., EE, ASD, and intellectual disability). We demonstrate that uncontrolled translation of *Elavl4* isoforms in RG and early neurons leads to defective development of glutamatergic neurons. Moreover, selective translation of distinct *Elavl4* isoforms via their alternative 5′ UTRs determines neuronal development. We also report that Celf1 is an upstream regulator of 5′ UTR-driven translation and plays a role in neuronal development through *Elavl4*. Finally, we provide the first evidence in both mouse and human neocortex that CELF1 and ELAVL4 proteins are markers of early and late ventral RG, respectively. Our work represents an advancement in understanding the neurodevelopmental mechanisms of RBP-regulated translation of distinct isoforms via alternative 5′ UTRs. Moreover, we provide evidence for the implications of this novel mechanism for neurodevelopment.

## Results

### RBP isoforms are translationally regulated during development

Our previous data has suggested that there are dynamic changes in transcript translation between E13 and E16 and especially of mRNA translation regulators^5,10,11,13^. Yet, the exact mRNAs and associated isoforms affected by these changes were unknown. To identify translationally-regulated isoforms, we performed an unbiased RNA-seq screen by polysome profiling of dissected wild type (WT) E13 (early neurogenesis) and E16 (mid-to-late neurogenesis) neocortices. We compared isoform levels in total mRNA and in translationally distinct fractions: 40S-60S-80S (including monosome) and polysomal fractions.

**Fig. 1:**
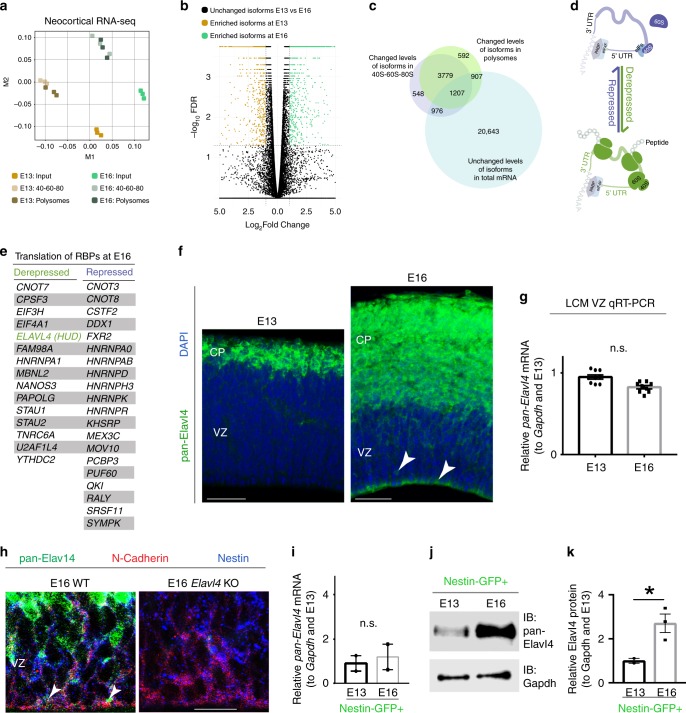
Elavl4 is translationally repressed during early neocortical neurogenesis. **a** Multidimensional scaling of neocortical tissue samples from E13 and E16 input (total RNA), 40S-60S-80S, and polysome fractions (*n* = 3 spins). Based on RNA-seq transcript abundances, M1 and M2 represent the first two dimensions. **b** Volcano plot of differentially expressed isoforms (gold and green dots) between E13 and E16 from input RNA-seq. Isoforms that are significantly higher at E13 are demarcated by gold circles, versus those higher at E16 are demarcated as green circles. Unchanged isoforms are demarcated as black circles. **c** Venn diagram of mRNAs that do not significantly change in their total expression levels (blue circle) compared to those that differ in association with 40S-60S-80S (purple circle), and/or polysomes (green circle). **d** Schematic: mRNA transcripts selected for further analysis were translationally repressed (top) or derepressed (bottom); these transcripts change from association with the 40S-60S-80S fraction (top) to the polysome fraction (bottom). **e** Translationally repressed and derepressed RNA-binding proteins (RBPs) at E16. **f** IHC for pan‐Elavl4 (green) in WT E13 and E16 neocortices (*n* = 12 animals per age). Arrowheads denote detectable Elavl4 signal in VZ at E16. DAPI shown in blue. Scale bar: 100 μm. **g** *pan-Elavl4* mRNA levels determined by qRT-PCR at E13 and 16 from LCM dissected VZ (*n* = 6 animals). Data represent the mean and SD. Data normalized to *Gapdh* and then to E13. Statistics: Student’s *t*-test. n.s. = not significant. **h** IHC for pan‐Elavl4 (green), N-Cadherin (red) and Nestin (blue) in WT and *Elavl4* KO E16 VZ (*n* = 3 animals). Arrowheads denote end feet. Scale bar: 50 μm. **i** Relative *pan-Elavl4* mRNA levels from FACS-sorted GFP+ cells determined by qRT-PCR (*n* = 3 FACS sorts per age and strain). Data represent the mean and SD. Data normalized to *Gapdh* and then to E13. Statistics: Student’s *t*-test. n.s. = not significant. **j** Western blot analysis of Elavl4-protein (top) from E13 and E16 Nestin-GFP+ FACS cells. Gapdh (bottom) was used as loading control. IB = immunoblotting. **k** Densitometry quantification of **j**. Data represent the mean and SEM. Data normalized to *Gapdh* and then to E13. Statistics: Welch’s *t*-test, 2-tailed, unpaired. *p < 0.05.

Multidimensional scaling revealed clusters distinguished by embryonic stage and fraction, confirming the reproducibility of replicates and the unique profiles of free and polysome-associated isoforms (Fig. 1a). The volcano plot of these data contains groups of isoforms that are potentially in diverse states of transcriptional or translational regulation (Fig. 1b). One group includes isoforms that are differentially expressed in their steady state levels, suggesting transcriptional regulation or changes in mRNA stability between E13 (Fig. 1b, gold dots) and E16 (Fig. 1b, green dots). The vast majority of isoforms showed steady state expression between E13 and E16 (30,073 isoforms; Fig. 1b, black dots). Most of these had comparable association with either monosome or polysome fractions (20,643 isoforms; Fig. 1c). However, within the 2114 isoforms that have steady state levels and altered their association with polysomes, 1143 mRNAs decreased association with polysomes between E13 and E16. We define these mRNAs as our set of potentially “translationally repressed mRNA isoforms” for further exploration (Fig. 1d, top). The 971 mRNA isoforms that were unchanged in total steady state levels but exhibited increased association with polysomes between E13 and E16 are defined as our set of “translationally derepressed mRNAs” (Fig. 1d, bottom).

To better understand the function of mRNAs that are translationally repressed or derepressed during neocortical neurogenesis, the 2114 steady state isoforms that change in their polysome association were further analyzed by gene ontology (GO) and KEGG using g:Profiler^26^. The terms enriched in repressed mRNAs were diverse in comparison to derepressed mRNAs (Supplementary Fig. 1). Enriched GO and KEGG groups included “RNA binding” and “Ribosome” in agreement with our previous report^27^. The clear enrichment of translational control pathways among translationally derepressed mRNAs at E16 implicated these mRNAs as being particularly dynamic between E13 and E16.

We also discovered several mRNAs encoding RBPs in our dataset to be translationally dynamic between E13 and E16 (Fig. 1e). Taken together, these findings reinforce the importance of precise translation control during neocortical development and especially as it proceeds from early to late neurogenesis^3,5,7,10,11,13,27^.

### Elavl4 is derepressed as neocortical neurogenesis proceeds

Among the translationally derepressed RBP mRNAs was *Elavl4*. Elavl4 is widely used as an early marker of post-mitotic neuronal differentiation and Elavl4-deficient embryonic neurospheres and adult subventricular zone progenitors demonstrate reduced capacity to differentiate into neuronal lineages^28,29^. However, the role of this RBP in prenatal neocortical development is unknown. We first established where Elavl4 protein was expressed at key time points during neurogenesis. Immunohistochemistry (IHC) using a pan-isoform antibody showed that Elavl4 is enriched in the cortical plate (CP) where post-mitotic neurons typically reside at E13 and E16 (Fig. 1f, left). Elavl4 was also enriched in the ventricular zone (VZ), where neocortical RG reside, at E16, but not at E13 (Fig. 1f, right). Using laser capture microdissection (LCM) of the VZ, we identified total *Elavl4* mRNA levels to be unchanged between E13 and E16 (Fig. 1g), supporting the idea that *Elavl4* is translationally derepressed between these two stages. Elavl4 colocalized with Nestin and N-Cadherin in RG end-feet in the E16 VZ (Fig. 1h, left, arrowheads). Elavl4 IHC was undetectable in Nestin and N-Cadherin-expressing RG in the VZ of *Elavl4* KO, demonstrating specificity of the antibody (Fig. 1h, right)^9,28^.

Since the developing neocortex has a heterogeneous cell population, we performed fluorescence-activated cell sorting (FACS) on *Nestin-GFP* transgenic mice to selectively isolate RG that typically express Nestin^30,31^ (Supplementary Fig. 2a). We also isolated intermediate progenitors and early neurons that typically express Tbr2 using *Tbr2-GFP* transgenic mice^32,33^. The mRNA levels of *pan*-*Elavl4* mRNA were unchanged between those two stages in both FACs populations (Fig. 1i; Supplementary Fig. 2b), mirroring the LCM results obtained in the entire VZ (Fig. 1g). However, Elavl4 protein significantly increased with time in Nestin-GFP^+^ RG (Fig. 1j, k) with an upward trend in Tbr2-GFP^+^ cells (Supplementary Fig. 2c). These data confirm that *Elavl4* is translationally derepressed as neurogenesis proceeds, especially in RG.

### Distinct 5′ UTRs dictate neocortical expression of Elavl4

**Fig. 2:**
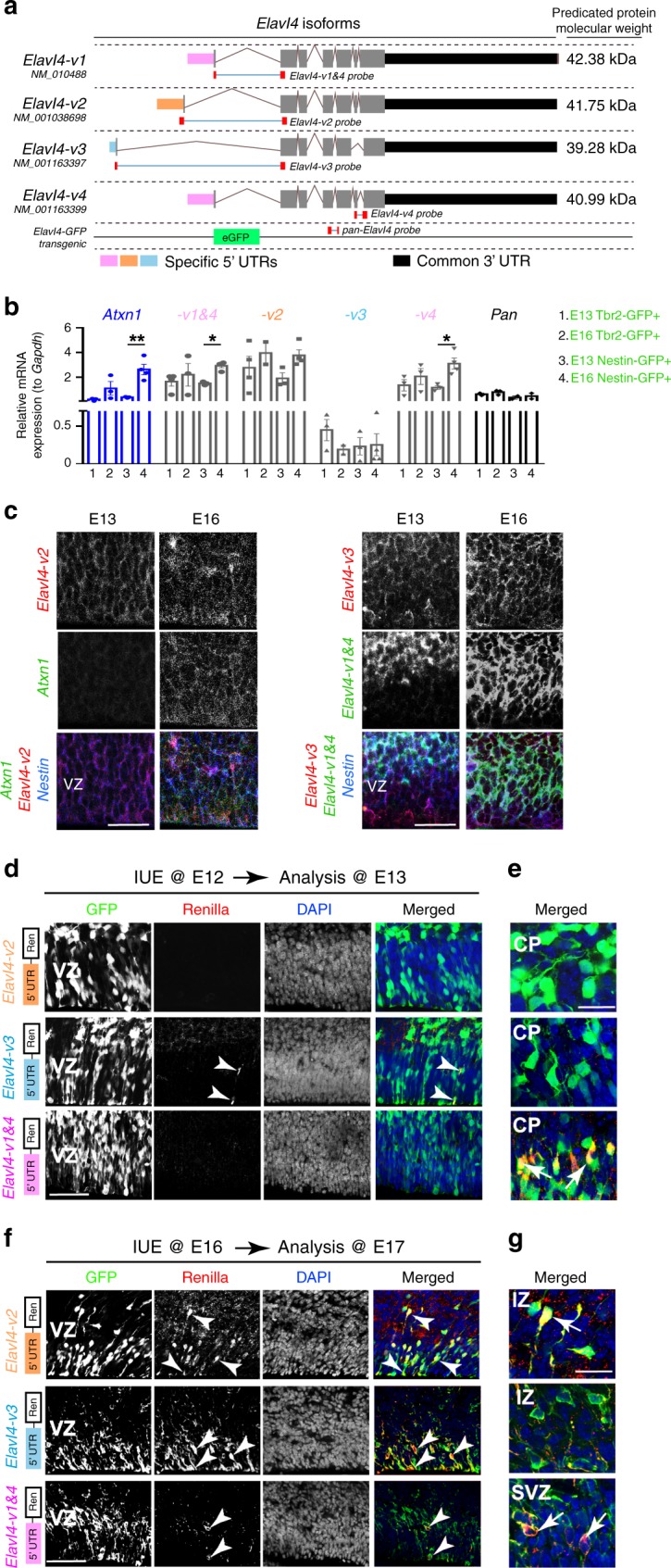
*Elavl4* isoforms are translationally derepressed in the VZ by mid-neurogenesis. **a** Schematic of the four *Elavl4* isoforms in mice. All isoforms share a common 3′ UTR sequence (black). Minor differences are in coding regions (exons in gray) and major differences in 5′ UTRs (distinguished by color). NCBI Accession numbers for each variant is indicated underneath common name and the location of qRT-PCR probes used is shown below (red). **b** Relative mRNA levels of positive control *Atxn1* and the different *Elavl4* isoforms from FACS-sorted Tbr2-GFP+ and Nestin-GFP+ cells determined by qRT-PCR (*n* = 3 FACS sorts per age). Data represent the mean and SEM. Data normalized to *Gapdh*. Statistics: Two-way ANOVA with Tukeyʼs. *p < 0.05. **c** Fluorescent in situ hybridization of E13 and E16 VZ for *Nestin* (blue), *Atxn1* (green, left), *Elavl4-v2* (red, left), *Elavl4-v3* (red, right), and *Elavl4-v4* (green, right). Scale bar: 20 μm. **d**, **f** E13 and E17 neocortices were *in utero* electroporated (IUE) at E12 (**d**) or E16 (**f**), respectively. VZ was transfected with CAG-GFP (green) and either *Elavl4-v2-5*′ *UTR-Renilla, Elavl4-v3-5*′ *UTR-Renilla* or *Elavl4-v1&4-5*′ *UTR-Renilla* (red) (*n* = 3 or 4 animals, respectively). The merged image is on the far right. Arrowheads represent colocalization in VZ. **e** and **g** show zoomed-in regions above VZ as indicated. Arrows represent colocalization in cortical plate (CP), intermediate zone (IZ) and subventricular zone (SVZ). DAPI is in blue. **d, f** scale bar: 40 μm. e, g scale bar: 20 μm.

*Elavl4* is spliced into 4 different isoforms in mice that have alternative 5′ UTRs and a common 3′ UTR (Fig. 2a). We took the advantage of the isoform-specific 5′ UTRs to differentiate between their expression in vivo using qRT-PCR (Fig. 2b). *Atxn1* mRNA is expressed in both progenitors and early neurons and was used as a positive control; its mRNA levels increased between E13 and E16 in GFP^+^ cells obtained from *Nestin-GFP* neocortices. *Elavl4-v2* and *-v3* steady state levels were maintained between the two FACS populations at both stages, while *Elavl4-v1&4* and *Elavl4-v4* increased in Nestin-GFP^+^ cells (Fig. 2b). We confirmed these findings with *Atxn1* and 5′ UTR -specific fluorescent in situ hybridization (FISH) probes in the E13 and E16 VZ (Fig. 2c). We further found increased association of distinct *Elavl4* isoforms with heavy polysomes at E16 compared to monosome or light polysome fractions (Supplementary Fig. 2d, e); *Elavl4-v3* could not be reliably measured given low expression. These findings corroborated E16 translational derepression of Elavl4, inferred from RNAseq data, and the divergence between mRNA and protein levels (Fig. 1). Collectively, these results indicate that precise translational control of distinct *Elavl4* isoforms occurs as neocortical neurogenesis proceeds.

We next aimed to understand Elavl4 isoform-specific protein expression. As there are no isoform-specific Elavl4 antibodies available, we co-transfected E12 and E16 RG with *CAG-GFP* and constructs bearing specific *Elavl4* isoform 5′ UTRs upstream of the *Renilla* coding sequence (Fig. 2d, f). In the VZ at E13, only sporadic *Elavl4-v3* 5′UTR-Renilla was expressed and no *Elavl4-v2* or *-v1&4* 5′ UTR-Renilla expression was observed (Fig. 2d). In contrast, *Elavl4-v1&4* 5′ UTR-Renilla was expressed in post-mitotic neurons (Fig. 2e), which was expected since Elavl4 is a known post-mitotic marker. *Elavl4-v2* and *-v3* 5′ UTR-Renilla were absent from post-mitotic neurons (Fig. 2e), suggesting that *Elavl4-v1&4* are the isoforms detected by the Elavl4 antibody in early neurons^9^. Indeed, we confirmed that the GFP in the *Elavl4-GFP* transgenic line previously used by our lab^9^ is inserted in place of *-v4’s* exon 1 (Fig. 2a) and is expressed in early post-mitotic neurons of developing neocortices (Supplementary Fig. 7d).

Remarkably, *Elavl4-v2* and *-v3* 5′ UTR-Renilla were abundantly expressed in the VZ at E17 (Fig. 2f), a stage when we detected Elavl4 protein in the VZ (Fig. 1f). *Elavl4-v1&4* 5′ UTR-Renilla was only sporadically observed in VZ mitotic cells at this time (Fig. 2f). *Elavl4-v3* 5′ UTR-Renilla expression was low or absent in migrating neurons in the intermediate zone (IZ), while *Elavl4-v2* and *-v1&4* 5′ UTR-Renilla remained expressed in neurons (Fig. 2g). These data are in line with the polysome fractionation experiment as well as with the expression of Elavl4 protein in the VZ, corroborating specificity of Elavl4 IHC. Utilizing this approach, we revealed isoform and cell-type specific regulation of *Elavl4* translation through its 5′ UTRs in the developing neocortex.

### *Elavl4* isoforms are bound by Celf1 in developing neocortices

**Fig. 3:**
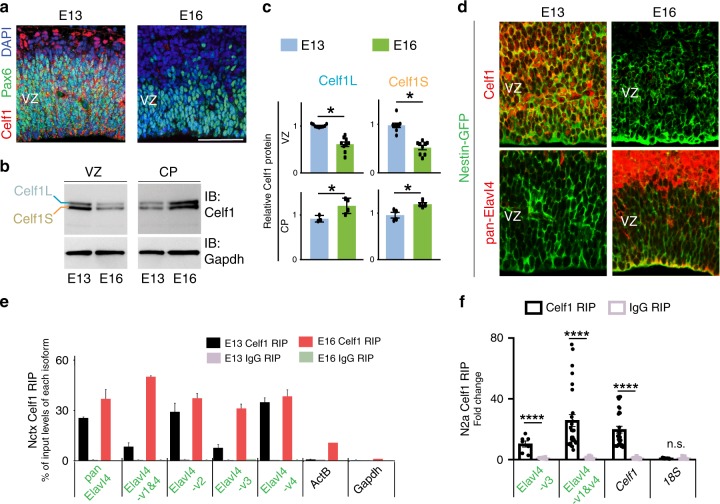
Celf1 binds *Elavl4* developing neocortices. **a** Celf1 (red) and Pax6 (green) VZ, IHC at E13 and E16 (*n* = 3 animals per age). DAPI shown in blue. **b** Western blot of Celf1 protein (upper panel) expression in VZ and CP from LCM neocortices at E13 and E16 (*n* = 3 animals per age). The Celf1 upper band is putatively Celf1 Long (Celf1L); the Celf1 lower band is putatively Celf1 Short (Celf1S), indicated at left. Gapdh was used as a loading control (bottom panel). **c** Densitometry quantification of **b**. Data represent the mean and SEM. Data normalized to *Gapdh* and then to E13. Statistics: Student’s *t*-test. *p < 0.05. **d** Celf1 (red, top) or Elavl4 (red, bottom) IHC on *Nestin-GFP* (green) neocortices at E13 and E16 (*n* = 4 animals per age). **e** RNA immunoprecipitation (RIP) from E13 and E16 neocortices. Relative mRNA levels were determined by qRT-PCR from Celf1 vs. IgG RIPs (*n* = 4 separate RIPs, 6 neocortices per age). *Pan-Elavl4* and specific *Elavl4* isoforms are shown in addition to *ActB* and *Gapdh* as non‐binding controls. **f** RIP from N2a cells (*n* = 3 RIPs from separate transfections). Data represent the mean and SEM. Data normalized to *Gapdh*. *Celf1* was used as positive control^60^. *18S* was used as negative control. Statistics: Two-way ANOVA with Tukeyʼs. ****p < 0.0001.

Based on these results, we hypothesized that a translational repressor acts on *Elavl4* at its isoform-specific alternative 5′ UTRs. We first tested whether the 5′ UTRs of all translationally repressed or derepressed mRNAs between E13 and E16 bear specific binding motifs (Supplementary Fig. 3a, b). We found the binding motif of RBP Celf1 to be present in 4.71% of the 5′ UTRs of translationally derepressed mRNAs, including *Elavl4s*′ (Supplementary Fig. 3a). *Celf1* mRNA expression has previously been reported in developing neocortices^5,22^. We observed that Celf1 protein is high in Pax6+ RG at E13 (Fig. 3a, left), but decreases by E16 (Fig. 3a, right). Western blot on LCM-dissected VZ and CP corroborated these observations in the VZ and showed an increase in Celf1 in the CP from E13 to E16 (Fig. 3b, c, Supplementary Fig. 3c). There was no IHC signal in *Celf1* KO VZ at E13 (Supplementary Fig. 3d), confirming the specificity of the Celf1 antibody. Celf1 colocalized with Nestin*-*GFP in the VZ at E13 and decreased by E16 contrasting Elavl4 IHC (Fig. 3d). The contrasting VZ expression patterns of Celf1 and Elavl4 support the hypothesis that Celf1 translationally represses *Elavl4* translation in vivo.

To assess whether Celf1 binds *Elavl4* isoforms, RNA immunoprecipitation (RIP) was performed on E13 and E16 neocortices using RIP-certified Celf1 antibody and corresponding IgG control (Fig. 3e). The resulting RNAs were assessed for the expression levels of all *Elavl4* isoforms along with negative controls (*β-actin* and *Gapdh*). Celf1 bound *Elavl4* transcripts in E13/E16 neocortices (Fig. 3e) at different levels compared to the input. These findings suggest that Celf1 may regulate *Elavl4* mRNA translation in developing neocortices on an isoform-specific level.

Therefore, our next goal was to understand how Celf1 acts on *Elavl4* mRNA, as RBPs can affect all steps of post-transcriptional processing from splicing to translation (Supplementary Fig. 4a). Celf1 exists in at least two isoforms: “long” representing the full-length protein and a “short” isoform with a 27 amino acid truncation at the N-terminus (Supplementary Fig. 4b, c). Functional differences between the two isoforms have not been previously established. Structurally, the RNA recognition motif 1 (RRM1) near the N-terminus is truncated in Celf1 short (Celf1S), while RRM3 near the C-terminus is shared between both isoforms. Comparison of RRM1^34^ and RRM3^35^ structures suggests their binding modes have meaningful differences.

**Fig. 4:**
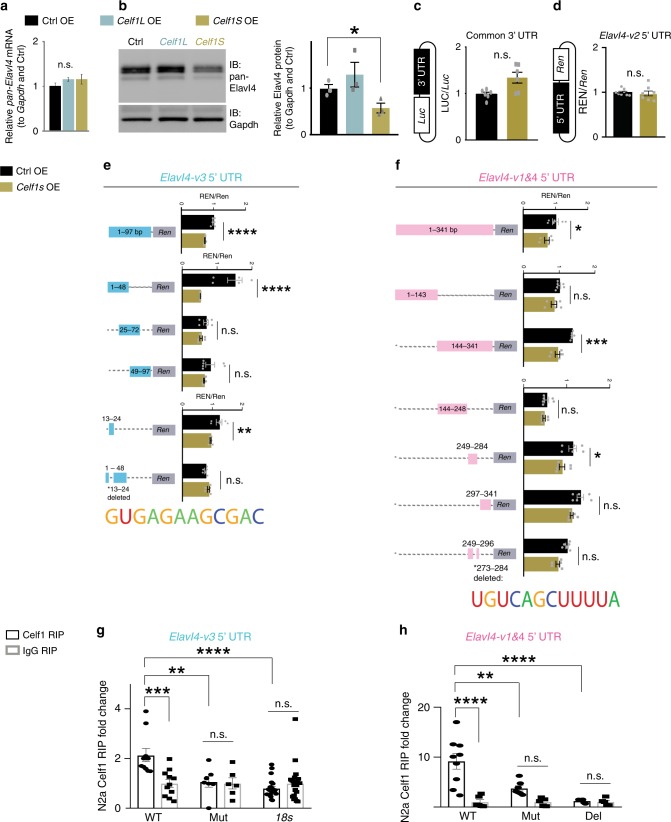
Celf1S translationally repressed *Elavl4-v3* and *-v4* via their 5′ UTRs. **a** Relative mRNA levels determined by qRT-PCR of *pan*-*Elavl4* after either Control (Ctrl), *Celf1L*, or *Celf1S* overexpression (OE) in N2a cells (*n* = 6 transfections). Data represent the mean and SEM. Data normalized to *Gapdh* and then to Ctrl. Statistics: one-way ANOVA. n.s. = not significant. **b** (left) Western blot for Ctrl, *Celf1L*, or *Celf1S* OE in N2a cells (*n* = 6 transfections). Immunoblot of pan-Elavl4 (upper panel). Gapdh is the loading control (lower panel). (right) Densitometry quantification of Western blots. Data represent the mean, SD. Data normalized to Gapdh and then to Ctrl. Statistics: one-way ANOVA. *p < 0.05. **c** Schematic of 3′ UTR luciferase construct (left of bar graph). *Elavl4* isoforms’ common 3′ UTR downstream of *Luciferase (Luc)*, and cotransfected with either Ctrl OE/*Celf1S* into N2a cells. Translation assayed by measuring LUC RLU over *Luc* mRNA levels (*n* = 9 transfections). Data represent the mean and SEM. Statistics: Student’s *t*-test. n.s. = not significant. **d** Schematic of 5′ UTR Renilla construct (left of bar graph). N2a cells were cotransfected with Ctrl OE or *Celf1S* and *Elavl4-v2* 5′ UTR upstream of *Renilla* (*Ren*). Same assay as in **c**; REN measured over *Ren* mRNA (*n* = 3 transfections). Data represent the mean and SEM. Statistics: Student’s *t*-test. n.s. = not significant. **e**, **f** Deletion strategy of Celf1-regulatory regions in 5′ UTRs of *Elavl4-v3* (**e**) and *Elavl4-v1&4* (**f**). *Elavl4-v3* or *Elavl4-v1&4* 5′ UTRs Renilla constructs; same strategy as in **d**. Dashed lines represent deleted regions. Numbers indicate positions in UTR, 5′ → 3′. *n* = 4 transfections for each experiment. bp = base pairs. Putative Celf1 regulatory sequence in *Elavl4* 5′ UTRs indicated under their respective bar graphs. Data represent the mean and SEM. Statistics: Student’s *t*-test and one-way ANOVA with Tukey’s (**e**, **f**). *p < 0.05; **p < 0.01; ***p < 0.001; ****p < 0.0001; n.s. = not significant. **g**, **h** Celf1 RIP of *Elavl4-v3* (**g**) and *Elavl4-v1&4* (**h**) 5′ UTRs. UTRs have WT, mutated (Mut), or deleted (Del) *Celf1* binding sequences. *18s* was used as negative control. Data represent the mean and SEM; normalized to *Gapdh*. *n* = 3 RIPs from 3 transfections; Statistics: Two-way ANOVA with Tukey’s. *p < 0.05, **p < 0.01, ***p < 0.001.

To investigate functional differences between the two isoforms, we turned to the Neuroblastoma (N2a) cell line for overexpression (OE) experiments. We first confirmed that Celf1 binds *Elavl4* in N2a cells (Fig. 3f). Upon OE of *Celf1L* or *Celf1S* (Supplementary Fig. 5a), there were no changes in *Elavl4* mRNA steady state levels (Fig. 4a, Supplementary Fig. 5b), splicing (Supplementary Fig. 5b), or decay (Supplementary Fig. 5d). However, *Celf1S* suppressed Elavl4 protein levels while *Celf1L* did not (Fig. 4b), suggesting that the shorter N-terminus imbues *Celf1S* with its repressive function. Combined with our RIP data, these results suggest that Celf1 suppresses *Elavl4* translation.

To test whether *Celf1* acts on *Elavl4* mRNA through the 3′ UTR or 5′ UTR, we cloned the 3′ UTR shared amongst all *Elavl4* isoforms downstream of *Luciferase* (*Luc*) and the 5′ UTRs specific to *Elavl4-v2*, *-v3*, *-v1&4* (the *Elavl4-v1* 5′ UTR is indistinguishable from the *-v4* 5′ UTR; Fig. 2a) upstream of *Renilla* (*Ren*). LUC or REN relative light units (RLU) represent protein expression levels and were normalized to *Luc* or *Ren* mRNA levels, respectively. The mRNA to protein ratio then serves as a proxy for translation level^11,13^. We found that *Celf1S* did not have an effect on translation mediated by the 3′ UTR (Fig. 4c) nor on *Elavl4-v2* 5′ UTR based translation (Fig. 4d). However, *Celf1S* suppressed translation of *Ren* via the *Elavl4-v3* and *-v1&4* 5′ UTRs (Fig. 4e, f; Supplementary Fig. 5f). We then sought to narrow down the effective regulatory region(s) in *Elavl4-v3* and *-v1&4* 5′ UTRs. Using the same translational assay and a series of deletion constructs, we identified the region between nucleotides 13-24 of *Elavl4-v3* 5′ UTR (Fig. 4e) and the region between nucleotides 273-284 of *Elavl4-v1&4* (Fig. 4f) from the 5′ UTR start site to be necessary for Celf1 regulation.

Celf1 binding sites in *Elavl4* 5′ UTRs were verified in a RIP experiment. When Celf1 binding sites were either deleted or mutated, the Celf1 binding of 5′ UTRs was significantly diminished (Fig. 4g, h). These data indicate that Celf1 can suppress *Elavl4* mRNA translation in an isoform-specific manner and through specific regulatory elements in the 5′ UTRs of *Elavl4-v3* and *-v1&4*.

### Celf1 silencing derepresses *Elavl4* translation

Robust translation occurs when multiple ribosomes (a polysome complex) simultaneously translate a single mRNA. If *Celf1S* OE results in decreased translation of the Elavl4 protein, then it is possible that some aspect of *Elavl4* polysome incorporation could be stymied in the presence of Celf1. Polysome fractionation under *Celf1S* OE in N2a cells did not yield significant shifts of *Elavl4-v4* from heavier to lighter fractions (Supplementary Fig. 5e), while *Elavl4-v3* was too low in polysome fractions to yield meaningful results. This suggests that excess Celf1 in our experiment is not sufficient to disturb *Elavl4* mRNA in polysomes.

**Fig. 5:**
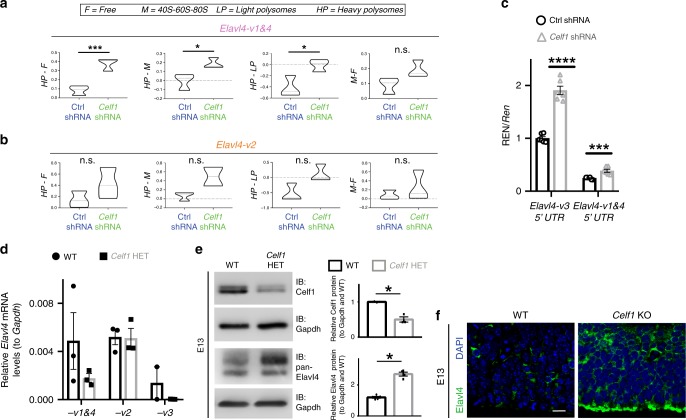
*Celf1* deletion derepressed *Elavl4-v3* and *Elavl4-v4* translation. **a**, **b** Polysome fractionation of N2a cells transfected with either Ctrl shRNA or *Celf1* shRNA. Difference between free (F), 40S-60S-80S monosome (M), light polysome (LP), and heavy polysome (HP) fractions are represented for *Elavl4-v1&4* (**a**) and *Elavl4-v2* (**b**). This graph plots mean difference for each condition; a higher mean difference means a greater abundance in the minuend (*n* = 3 fractionations, each fractionation was from one 10 cm plate transfection). Statistics: Welch’s *t*-test. ***p < 0.001. *p < 0.05, n.s. = not significant. **c**, N2a cells cotransfected with Ctrl shRNA or *Celf1* shRNA plasmids and *Elavl4-v3* or *Elavl4-v1&4-5*′ *UTR*s cloned upstream of Renilla. Same assay as in Fig. 4. Multiple T-tests. *p < 0.05, ***p < 0.001. **d** qRT-PCR determined levels of different *Elavl4* isoforms in E13 WT and *Celf1* HET neocortices. Data represent the mean and SEM. *N* = 3 separate spins with 6 brains per spin. Data normalized to *Gapdh*. **e** (left) Western blot for WT and *Celf1* HET neocortices at E13 (*n* = 9 neocortices). (right) Densitometry quantification of Western blots. Data represent the mean and SD. Data normalized to Gapdh and then to WT. Statistics: Student’s *t*-test. *p < 0.05. **f** Neocortical VZ at E13 of WT (*n* = 6 animals) and *Celf1* KO (*n* = 2 animals). IHC for pan-Elavl4 (green). DAPI shown in blue. Scale bar: 40 μm.

Meanwhile, in the *Celf1* knockdown condition in N2a cells (Supplementary Figs. 4d, 6a), *Elavl4-v1&4* isoform presence on heavy polysomes increased relative to free, 40S-60S-80S monosome, and light polysome fractions relative to control (Ctrl) shRNA (Fig. 5a). *Elavl4-v2* did not shift significantly (Fig. 5b) and again *Elavl4-v3* unreliably amplified. This aligns with increased translation of *Ren* via the *Elavl4-v1&4* 5′ UTR in *Celf1* knockdown (Fig. 5c). In addition, we found increased translation of *Ren* via the *Elavl4-v3* 5′ UTRs in *Celf1* knockdown (Fig. 5c). Silencing *Celf1* in N2a cells elevated steady-state levels of *Elavl4* mRNAs (Supplementary Fig. 6d, f, h), but there was no change in splicing nor in decay (Supplementary Figs. 5b, 6c, e, g). Collectively, our results indicate that a decrease in Celf1 derepresses *Elavl4* mRNA translation.

To determine the effect of Celf1 on Elavl4 in vivo, we compared E13 WT mice to *Celf1* heterozygous (*Celf1* HET) littermates. *Celf1* HETs were analyzed because global deletion of *Celf1* in mice reduces viability in a background-strain dependent manner^36,37^. While steady state levels of *Elavl4-v2* did not change, *Elavl4-v1&4* and *-v3* mRNA decreased in *Celf1* HET (Fig. 5d), contrasting the increase in Elavl4 protein expression at E13 (Fig. 5e). Low amounts of mRNAs paralleled by a high level of corresponding protein expression has been reported in multiple systems^3,10,11,38,39^. From the *Celf1* KO neocortices that we managed to obtain, Elavl4 protein was enriched in the VZ when compared to WT at E13 (Fig. 5f), suggesting that Celf1 suppresses *Elavl4* mRNA translation in vivo during early neocortical neurogenesis.

### *Elavl4-v3* promotes lower layer (LL) identity when not repressed in RG

**Fig. 6:**
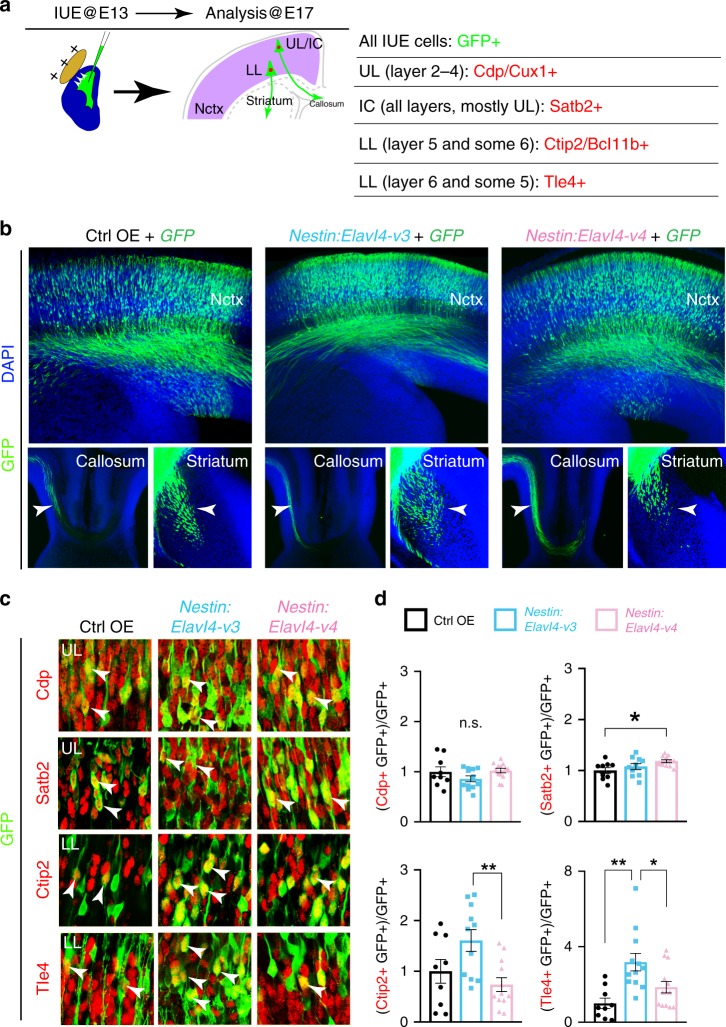
*Elavl4-v3* OE lacking its 5′ UTR in neural stem cells promotes LL identity. **a** Schematic: Neocortical IUE at E13 (left) with *CAG-GFP* and either Ctrl or *Elavl4* constructs were analyzed at E17 for colocalization between GFP and neocortical layer markers as indicated in the chart on the right. Nctx = neocortex, UL = upper layers, LL = lower layers, IC = intracortically projecting. **b** Representative mages of E17 IUE Nctx, corpus callosum, and striatum. GFP+ axons (green) are indicated by arrowheads. Scale bar: 200 μm. **c** Representative images of immunostained E17 IUE Nctx electroporated with GFP and either Ctrl (*n* = 3 animals), *Nestin:Elavl4-v3* (*n* = 4 animals), or *Nestin:Elavl4-v4* (*n* = 4 animals) OE. IHC for GFP (green) and cortical neuron identity markers (red) listed in **a**. Scale bar: 20 μm. **d** Quantification of colocalization between layer markers and GFP, normalized to Ctrl. Data represent the mean of sections and SEM. Statistics: one-way ANOVA with Tukey’s. *p < 0.05, **p < 0.01, ***p < 0.001.

To determine the consequence of premature *Elavl4* derepression in early neocortices, we cloned *Elavl4-v3* and *-v4* without their native 5′ UTRs downstream of the RG-specific *Nestin* promoter. Ctrl, *Elavl4-v3*, and *-v4* OE constructs were *in utero* electroporated (IUE) with* CAG-GFP* into E13 RG (Fig. 6a). GFP^+^ axons projected appropriately for the developmental stage in each condition (Fig. 6b). IHC of transfected neocortices at E17 revealed that when *Elavl4-v3* was not translationally repressed in RG, there was an increase in Tle4^+^ and an upward trend in Ctip2^+^ LL identity compared to Ctrl (Fig. 6c, d). This finding is in line with our previous report that postnatal Tle4^+^ neuronal identity is reduced by *Elavl4* deletion^9^. There was no effect on Cdp^+^ upper layer (UL) neuronal identity by E17 when *Elavl4-v3* is OE in early RG (Fig. 6d). However, premature *Elavl4-v4* expression in RG resulted in a slight increase of Satb2^+^ intracortically (IC) projecting neurons and a trending decrease of Ctip2^+^ LL neurons when compared to Ctrl or *Nestin:Elavl4-v3*, respectively (Fig. 6c, d). These data indicate that premature expression of *Elavl4-v3* in RG results in a misbalance of neocortical glutamatergic neurons.

### *Elavl4-v3 or -v4* with native 5′ UTRs promote UL identity

**Fig. 7:**
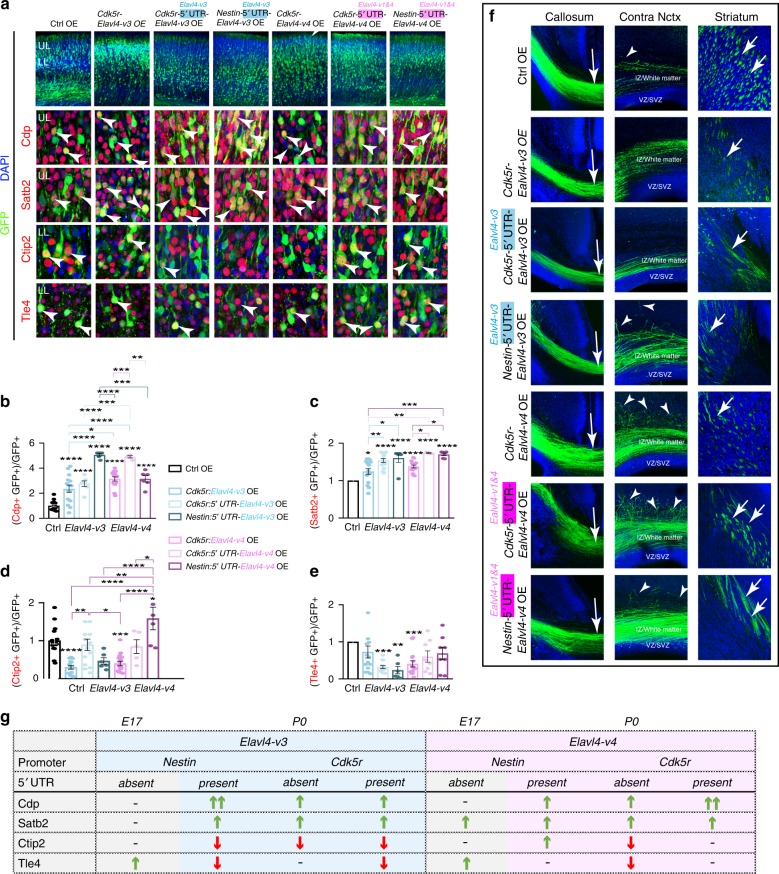
Native 5′ UTRs of *Elavl4* isoforms dictate neocortical glutamatergic identities. **a** (top) P0 representative confocal images of E13 IUE neocortices electroporated with *CAG-GFP* and either Ctrl (*n* = 6 animals), *Cdk5r:Elavl4-v3* (*n* = 5 animals), *Cdk5r:5*′ *UTR-Elavl4-v3* (*n* = 4 animals), *Nestin:5*′ *UTR-Elavl4-v3* (*n* = 3 animals), *Cdk5r:Elavl4-v4* (*n* = 6 animals), *Cdk5r:5*′ *UTR-Elavl4-v4* (*n* = 3 animals), *Nestin:5*′ *UTR-Elavl4-v4* (*n* = 3 animals) OE plasmids. IHC for GFP (green), and cortical layer identity markers Cdp, Satb2, Ctip2, or Tle4 (all red). Scale bar: 20 μm. DAPI shown in blue. **b**–**e** Quantification of colocalization between layer markers (arrowheads in A) and GFP normalized to controls. Data represent the mean of sections and SEM. Statistics: one-way ANOVA with a post-hoc Tukeyʼs. *p < 0.05, **p < 0.01, ***p < 0.001, ****p < 0.0001. All significance stars that are located directly above bars are in reference to the Ctrl, otherwise indicated with line above. **f** P0 representative confocal images of E13 IUE neocortices electroporated with GFP and either Ctrl (*n* = 6 animals), *Cdk5r:Elavl4-v3* (*n* = 5 animals), *Cdk5r:5*′ *UTR-Elavl4-v3* (*n* = 4 animals), *Nestin:5*′ *UTR-Elavl4-v3* (*n* = 3 animals),*Cdk5r:Elavl4-v4* (*n* = 6 animals), *Cdk5r:5*′ *UTR-Elavl4-v4* (*n* = 3 animals), *Nestin:5*′ *UTR-Elavl4-v4* (*n* = 3 animals) OE plasmids IHC for GFP (green). Arrows indicate GFP+ axons passing through corpus callosum and striatum ipsilateral to IUE site. Arrowheads indicate GFP+ axons growing into neocortex contralateral to IUE site. DAPI shown in blue. Scale bar: 200 μm. **g** Summary table of results in Fig. 6 (E17; gray) and 7a-7e (P0; colored). Increased colocalization is indicated with a green up arrow and decreased colocalization is indicated with a red down arrow.

We next tested whether neuronal identities are disrupted when *Elavl4* isoforms are aberrantly expressed in neurons. *Elavl4-v3* and *-v4* were cloned under the *Cdk5r* promoter which is selectively expressed in early post-mitotic neurons^40^. Ctrl, *Cdk5r:Elavl4-v3*, or *Cdk5r:Elavl4-v4* OE were IUE with *CAG-GFP* into RG at E13. Analysis at P0 revealed strikingly different results than the OE experiments in RG. When either *Elavl4-v3* or *-v4* are OE in early neurons, there was an increased number of UL Cdp^+^ and IC Satb2^+^ neuronal identities compared to Ctrl (Fig. 7a–c, g). These data suggest that the aberrant expression of *Elavl4* in post-mitotic neurons promotes UL identities.

To determine if these effects were due to the lack of the native 5′ UTRs, we added isoform-specific 5′ UTRs to the cell-type specific promoter-driven constructs used thus far. Constructs were IUE with *CAG-GFP* into E13 RG. Appropriate expression of each 5′ UTR construct was confirmed using a specific FISH probe (Supplementary Fig. 7a). Surprisingly, while the UL Cdp+ identity was not affected by *Elavl4-v3* OE in RG (Figs. 6d and 7g), the addition of the native 5′ UTR in RG increased this subpopulation (Fig. 7b, g; *Nestin:5*′ *UTR-Elavl4-v3*). UL Cdp+ and IC Satb2+ identities were increased after OE of either *v-3* or *-v4* isoform in neurons. This effect was potentiated by the presence of 5′ UTR in RG for *Elavl4-v3* (*Nestin:5*′ *UTR-Elavl4-v3*). Meanwhile, the presence of the native *Elavl4-v4* 5′ UTR potentiated the increase of UL Cdp+ and IC Satb2+ identities only when expressed in neurons (*Cdk5r:5*′ *UTR-Elavl4-v4*), but not in RG (*Nestin:5*′ *UTR-Elavl4-v4*). These results indicate that *Elavl4* isoforms promote UL and IC identity when the 5′ UTR is present.

### LLs are sensitive to the presence of distinct *Elavl4* 5′ UTRs

We next turned our focus to the effect of 5′ UTRs on LL identities. LL Ctip2^+^ identity decreased regardless of 5′ UTR presence when *Elavl4-v4* was expressed in neurons (Fig. 7d, g; *Cdk5r:Elavl4-v4* and *Cdk5r:5*′ *UTR-Elavl4-v4*). *Elavl4-v3* OE in RG decreased LL Ctip2^+^ identity only when its native 5′ UTR was present (Fig. 7d, g; *Nestin:5*′ *UTR-Elavl4-v3*). *Elavl4-v4* OE in neurons also decreased LL Ctip2^+^ identity (Fig. 7d, g; *Cdk5r:Elavl4-v4*). However, this isoform again proved to be especially sensitive to the presence of its 5′ UTR. When the *Elavl4-v4* is OE in neurons with its 5′ UTR, the LL Ctip2^+^ identity was normalized (Fig. 7d, g; *Cdk5r:5*′ *UTR-Elavl4-v4*). Moreover, when the *Elavl4-v3* is introduced in the RG with its 5′UTR, the LL Ctip2^+^identity increased (Fig. 7d, g; *Nestin:5*′ *UTR-Elavl4-v3*).

The LL Tle4^+^ subpopulation was likewise sensitive to isoform and 5′ UTR presence. Even though both *Elavl4-v3* and *-v4* promoted Tle4^+^ identity when expressed in RG (Figs. 6d and 7g; *Nestin:Elavl4-v3* and *Nestin:Elavl4-v4*), *Elavl4-v3* decreased this identity in the presence of its 5′ UTR in both RG and neurons (Fig. 7e, g; *Nestin:5*′ *UTR-Elavl4-v3* and *Cdk5r:5*′ *UTR-Elavl4-v3*). In contrast, by the presence of *Elavl4-v4* 5′ UTR in either RG or neurons, Tle4^+^ identities were unaffected (Fig. 7e, g; *Nestin:5*′ *UTR-Elavl4-v4* or *Cdk5r:5*′ *UTR-Elavl4-v4*). This indicates that translational regulation of *Elavl4-v4* leads to the appropriate balance of the Tle4^+^ identity (Fig. 7g). Surprisingly, the different constructs also appeared to have differential effects on migration (Fig. 7a, top row, Supplementary Fig. 9a, b).

GFP+ axons in all experimental conditions crossed the corpus callosum (Fig. 7f) and followed the white-matter tract above the VZ/SVZ^41^. However, differences were observed in the contralateral hemisphere of the IUE (Fig. 7f). We observed axons turning and growing into the opposite hemisphere when the 5′ UTR was present in the OE constructs. This defect was particularly robust when the *Nestin* promoter was driving *Elavl4-v3* (*Nestin:5*′ *UTR-Elavl4-v3*) and when *Cdk5r* was driving *Elavl4-v4* (*Cdk5r:5*′ *UTR-Elavl4-v4*) (Fig. 7f). This finding suggests the excessive outgrowth when overexpression was restricted to the appropriate cell types (*Elavl4-v3* in RG and *Elavl4-v4* in neurons). When compared to Ctrl OE, fewer GFP^+^ axons reached the striatum in all conditions except when *Elavl4-v4* OE was regulated by its 5′ UTR (Fig. 7f; *Nestin:5*′ *UTR-Elavl4-v4* and *Cdk5r:5*′ *UTR-Elavl4-v4*). It should also be noted that GFP^+^ cells from FACS-sorted transgenic lines co-express *Cdp* and *Tle4* mRNA (Supplementary Fig. 8c), supporting the importance of translational control and corroborating recent findings that RG are transcriptionally primed^15^. These data corroborate the idea that translational regulation of distinct *Elavl4* isoforms in early post-mitotic neurons strongly impacts the development of glutamatergic neurons to the risk of neuroanatomical pathologies.

### Celf1 regulates the balance of glutamatergic neurons

Since Celf1 translationally repressed *Elavl4* isoforms through their 5′ UTRs, we tested if mutating Celf1 binding sites in *Elavl4* 5′ UTRs abolished the defects observed in Fig. 7. We IUE *CAG-GFP* and either Ctrl OE, *5*′ *UTR-Elavl4-v3, 5*′ *UTR-Elavl4-v4*, or Celf1 binding site (BS)-mutated constructs (*Celf1BSmut:5*′ *UTR-Elavl4-v3* and *Celf1BSmut:5*′*UTR-Elavl4-v4*) at E13 and analyzed transfected neocortices at P0. The mutation of Celf1 BS in 5′ UTRs of *Elavl4* isoforms abolished the effects on UL Cdp^+^ and LL Ctip2^+^ identities induced by native 5′ UTRs (Supplementary Fig. 9c, d).

**Fig. 8:**
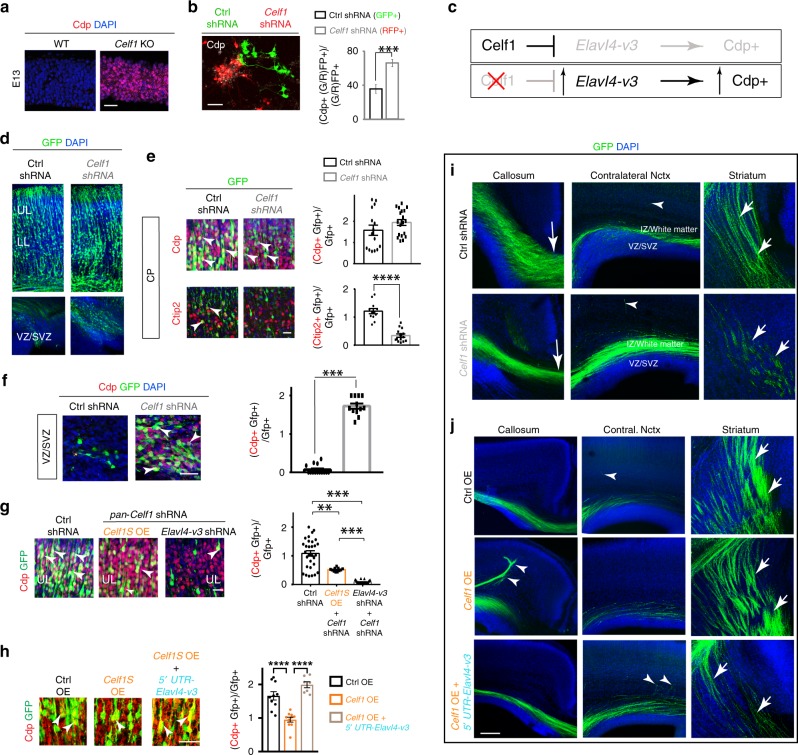
Celf1 regulates development of glutamatergic neurons in developing neocortices through *Elavl4*. **a** Neocortical E13 VZ of WT (*n* = 6 animals) and *Celf1* KO (*n* = 2 animals). IHC for Cdp (red). DAPI shown in blue. Scale bar: 100 μm. **b** (left) Representative IHC of three days in vitro primary neocortical neuronal cultures. Transfected neurons are expressing GFP (Ctrl shRNA) or RFP (*Celf1* shRNA) and neuronal identity marker Cdp (white). Scale bar: 40 μm. (right) Quantification of Cdp colocalization. Data represent the mean and SEM. *N* = 1 litter (14 embryos). Statistics: Students *t*-test. ***p < 0.001. **c** Schematic: Celf1 translationally represses *Elavl4-v3*, which decreases Cdp+ identities. Once Celf1 is silenced, *Elavl4-v3* translation is derepressed, increasing Cdp+ identities. **d** P0 representative confocal images of E13 IUE neocortices electroporated with *CAG-GFP* and either Ctrl shRNA (*n* = 5 animals) or *Celf1* shRNA (*n* = 4 animals) at E13. Ventricular zone/subventricular zone (VZ/SVZ) shown underneath. GFP is green. DAPI shown in blue. Scale bar: 100 μm. UL = upper layers, LL = lower layers. **e** (left) P0 representative confocal images of transfected neocortices in **d**, immunostained for cortical neuronal identity markers Cdp and Ctip2 in CP. Arrowheads indicate colocalization. Scale bar: 40 μm. (right) Quantification of Cdp and Ctip2 colocalization. Data represent the mean and SEM. Statistics: Student’s *t*-test. ***p < 0.001. **f** (left) Representative confocal images of transfected neocortices in **e**, immunostained for cortical neuronal identity marker Cdp in VZ/SVZ. Arrowheads indicate colocalization. Scale bar: 40 μm. (right) Quantification of Cdp colocalization. Data represent the mean of sections and SEM. Statistics: Student’s *t*-test. ***p < 0.001. **g** (left) P0 representative confocal images of E13 IUE neocortices electroporated with *CAG-GFP* and either Ctrl shRNA (*n* = 5 animals), *Celf1* shRNA and *Celf1* OE (*n* = 3 animals), or *Celf1* shRNA and *Elavl4-v3* shRNA (*n* = 3 animals). IHC for GFP (green) and layer marker Cdp (red). Arrowheads indicate colocalization. Scale bar: 40 μm. UL = upper layer. (right) Quantification of Cdp colocalization. Data represent the mean of sections and SEM. Statistics: one-way ANOVA with Tukeyʼs. *p < 0.05, ***p < 0.001, n.s. = not significant. **h** (left) P0 representative confocal images of E13 IUE neocortices electroporated with GFP and either Ctrl (*n* = 4 animals), *Celf1* OE (*n* = 3 animals), or *Celf1* OE and *5*′ *UTR-Elavl4-v3* (*n* = 3 animals) OE plasmids IHC for GFP (green), and cortical layer identity markers Cdp (red). Arrowheads indicate colocalization. DAPI shown in blue. Scale bar: 40 μm. (right) Quantification of Cdp colocalization. Data represent the mean and SEM. Statistics: one-way ANOVA with Tukeyʼs. ***p < 0.001. **i**, **j** Representative confocal images of the corpus callosum, contralateral neocortex, and striatum of P0 neocortices that underwent IUE with GFP and either Ctrl shRNA (n = 5 animals) or *Celf1* shRNA (*n* = 4 animals) (**i**) or either Ctrl OE (*n* = 3 animals), *Celf1* OE (*n* = 3 animals), or *Celf1* OE and *5*′ *UTR-Elavl4-v3* (**j**). Scale bar is 200 μm.

To assess Celf1’s possible role on identities, we first analyzed *Celf1* HET and KO neocortices at E13 when Elavl4 protein is increased. *Celf1* deletion increased Cdp^+^ and decreased Ctip2^+^ identity (Fig. 8a, Supplementary Fig. 7c–f), mirroring *Elavl4* OE phenotypes. To interrogate the role of Celf1 cell-autonomously, we performed IUE electroporation of Ctrl shRNA+*CAG-GFP* or *Celf1* shRNA+*CAG-RFP* into separate embryos of the same litter and performed in vitro analysis using a previously reported approach^13^ (Supplementary Fig. 8a). After three days in vitro (DIV) we found that Cdp^+^ identity increased (Fig. 8b) and Ctip2^+^ identity decreased in *Celf1* knockdown (Supplementary Fig. 8b). These in vitro findings substantiated the observation in the *Celf1* HET and KO neocrtices (Fig. 8a, Supplementary Fig. 7c–f). Notably, the identity changes observed in *Celf1-*depleted neocortices and neurons is comparable to *Elavl4-v3* and *-v4* OE without their 5′ UTRs (Fig. 7).

The possible *Celf1* HET effects on identities are independent of the cell cycle, Tbr2+ progenitors, or neurogenesis (Supplementary Fig. 7b). However, we found a higher retention of RG Pax6^+^ progenitors in *Celf1* HET neocortex (Supplementary Fig. 7b). Since Elavl4 protein is increased in *Celf1* HETs, the Pax6^+^ retention corroborates previous findings where *Elavl4* decrease resulted in fewer progenitors^28,29^. Overall, these data suggest that Celf1 acts on the balance of glutamatergic neurons through Elavl4.

### Celf1 regulates development of the neocortex via *Elavl4*

The data thus far suggest that *Celf1* reduction leads to a selective neuronal identity misbalance, possibly induced by Elavl4 increase (Fig. 8c). We next IUE E13 RG with *CAG-GFP* and either Ctrl or *Celf1* shRNA and analyzed neocortices at P0. *Celf1* silencing increased the number of transfected cells within the VZ/SVZ region (Fig. 8d, bottom), possibly due to a migration defect. Upon examination of neurons that reached the CP, there was no difference in UL Cdp^+^ identity between Ctrl and *Celf1* shRNA, while LL Ctip2^+^ identity in the CP decreased (Fig. 8e). However, when we analyzed GFP^+^ cells in the VZ, UL Cdp^+^ identity increased when *Celf1* was silenced (Fig. 8f). Co-expression of *Celf1* shRNA and *Celf1* OE decreased the UL Cdp^+^ identity (Fig. 8g), suggesting the specificity of *Celf1* shRNAs.

To test if increased UL Cdp^+^ identity after *Celf1* silencing is due to *Elavl4-v3* (Fig. 8c), we obtained specific *Elavl4-v3* shRNAs (Supplementary Fig. 4f). *Elavl4-v3* shRNA decreased UL Cdp^+^ identities and this was rescued by co-IUE with human *ELAVL4-V3* OE that is resistant to the mouse-specific shRNA (Supplementary Fig. 8f). Remarkably, silencing *Elavl4-v3* in *Celf1-*depleted neurons decreased UL Cdp^+^ identities (Fig. 8g) initially induced by *Celf1* silencing (Fig. 8a, b, f). Taken together, these data suggest that *Elavl4-v3* is downstream to *Celf1* in the regulation of neocortical neuronal identities and their development.

To further investigate the pathway, we IUE E13 neocortices with *CAG-GFP* and either Ctrl or *Celf1S* OE and analyzed the transfected neocortices at P0. *Celf1S* OE decreased Cdp^+^ identity (Fig. 8h). The *Celf1S* OE effect on the UL Cdp^+^ identity was reversed when *5*′ *UTR-Elavl4-v3* (Supplementary Fig. 4e) was co-IUE (Fig. 8h). Interestingly, co-IUE of *5*′ *UTR-Elavl4-v3* and *Celf1S* OE rescued UL Cdp^+^ identity up to control levels. This rescue further indicates a suppressive upstream role of *Celf1S* on *Elavl4* phenotypes.

When *Celf1* was silenced in vivo, Tle4^+^ identity decreased (Supplementary Fig. 8e). *Celf1S* OE in *Celf1* shRNA cells partially rescued Tle4^+^ identity and silencing of *Elavl4-v3* in *Celf1* shRNA cells reversed the Tle4^+^ identities (Supplementary Fig. 8e), further corroborating that *Elavl4-v3* is downstream to *Celf1*. While Celf1 silencing can promote Tle4^+^ identity in vitro, these data indicate that Tle4^+^ identity is indirectly influenced by Celf1 in vivo, possibly non cell-autonomously.

When *Celf1* was silenced, GFP+ axons were found to cross the CC and accumulate above the VZ/SVZ. Meanwhile, a considerably reduced number of GFP+ axons reached the striatum (Fig. 8i). This axonal distribution matched the increase in Cdp^+^ and decrease in Ctip2^+^ neurons projecting through callosum or subcortically, respectively (Fig. 8b, e, f, Supplementary Fig. 8b). Remarkably, *Celf1S* OE induced an aberrant GFP^+^ tract to terminate ipsilaterally to the IUE instead of crossing the corpus callosum (Fig. 8j). The abnormal ipsilateral tract was rescued when *Celf1S* was co-OE with *5*′ *UTR-Elavl4-v3* (Fig. 8j). All in all, these data indicate that Celf1 regulates development of glutamatergic neurons through *Elavl4*. Given that Celf1 OE at E13 affected connectivity, we IUE E16.5 neocortices with *CAG-RFP*+ Ctrl OE in one hemisphere and *CAG-GFP*+ *Celf1S* OE in the contralateral hemisphere and analyzed neocortices at P7. Remarkably, the hemisphere with *Celf1S* OE had ectopic GFP^+^ subcortical projections descending through the striatum and reaching the internal capsule (Supplementary Fig. 9e). In the contralateral RFP^+^ Ctrl hemisphere no axons were observed in the thalamus or the internal capsule. These data further suggest that *Celf1S* OE can disrupt callosal development and induce ectopic subcortical projections from IC-projecting neurons.

### CELF1 and ELAVL4 are expressed in human ventral RG

**Fig. 9:**
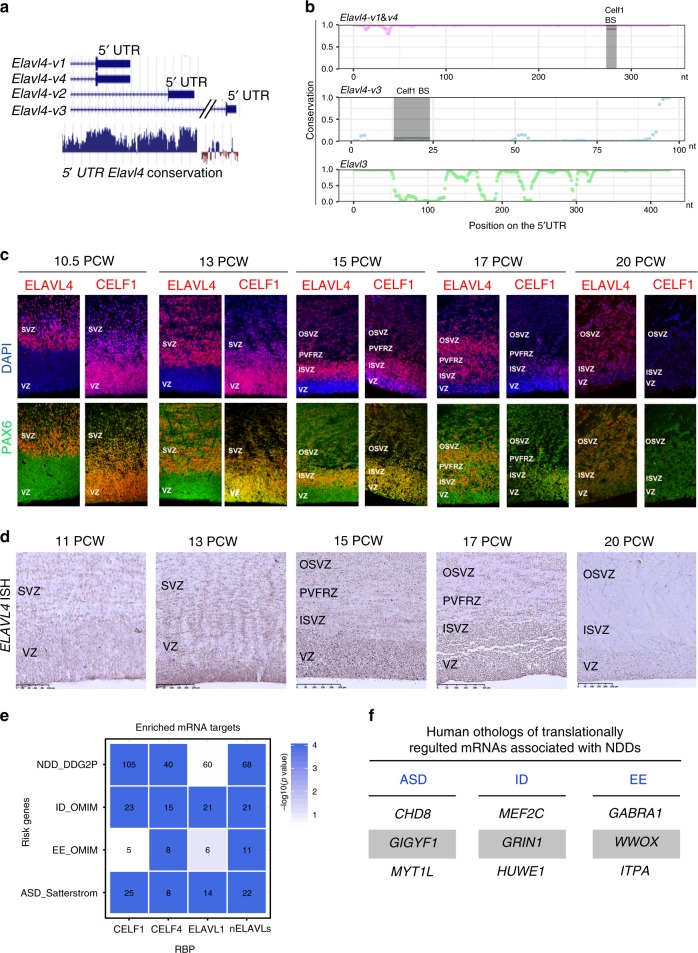
ELAVL4 and CELF1 have opposing expression in developing human neocortical VZ. **a** Alignment of mouse *Elavl4* 5ʼ UTRs (top). Conservation of UTRs shown below. **b** The plot shows the probability (y-axis) that each nucleotide in the 5ʼ UTR (x-axis) belongs to a conserved element for *Elavl4-v1/4* (pink), *Elavl4-v3* (light blue) and Elavl3 (green). Each dot represents a base pair. Celf1 regulatory binding sites (BS)-are indicated by the black bar/shaded area. nt, nucleotide. **c** Representative confocal images of human ventricular zone (VZ), inner subventricular zone (ISVZ), periventricular fiber rich zone (PVFRZ), and outer subventricular zone (OSVZ) at 10.5, 13, 15, 17, and 20 PCW in the frontal lobe. IHC for PAX6 (green) and either CELF1 (red) or ELAVL4 (red), indicated above image. DAPI shown in blue. **d** Representative images of in situ hybridization (ISH) for *Elavl4* on human VZ, ISVZ, PVFRZ, OSVZ at 11, 13, 15, 17, and 20 PCW in the frontal lobe. Scale bar: 100 μm. **e** The heatmap shows the degree of enrichment for mRNA targets of neuronal ELAVLs (nELAVLs), ELAVL1, CELF1 and CELF4 across neurodevelopmental disorders genes defined by the Developmental Disorders Genotype-Phenotype Database (NDD_ DDG2P), epileptic encephalopathy genes in OMIM (EE_OMIM), intellectual disability genes in OMIM (ID_OMIM) and ASD risk genes (PMID: 31981491). Color coding indicates –log10(p-value) after 10,000 permutations. Number of overlapping genes is indicated in each cell. **f** Examples of top translationally regulated genes implicated with ASD, ID, and EE. Note that some of these genes can manifest with co-morbidities.

Given the striking impact of Celf1-mediated regulation on identity and connectivity of glutamatergic neurons, we examined if the 5′ UTRs of mouse *Elavl4* isoforms were conserved in human. At least fifteen human *ELAVL4* isoforms exist (https://genome.ucsc.edu/), suggesting an increase in the complexity of translational control throughout evolution. The 5′ UTR of mouse -*v1&4* was highly conserved across species, including the region of Celf1 regulatory site (Fig. 9a, b). Meanwhile, the 5′ UTRs of mouse *-v3* and *Elavl3* (HuC) were less conserved (Fig. 9b), suggesting evolutionary divergence of the ELAVL4 protein.

We next wanted to determine if the CELF1 and ELAVL4 protein expression patterns in human developing neocortices resemble those we found in mice. We found conserved changes in ELAVL4 and CELF1 expression in the VZ and SVZ as human neocortical neurogenesis proceeds (Fig. 9c). ELAVL4 protein was enriched in the ventral RG of the VZ at later stages of neurogenesis, 17 and 20 post-conceptual weeks (PCW), which was decreased at 10 and 13 PCW. *ELAVL4* mRNA has been reported to be expressed ventral RG and as a novel human intermediate progenitor marker in human developing neocortices between 14 and 16 PCW^42,43^. In agreement with this, we found ELAVL4-protein expression in the inner subventricular zone (ISVZ) and outer subventricular zone (OSVZ) of all analyzed stages, but also scattered across the periventricular fiber rich zone (PVFRZ) positioned above VZ and ISVZ^44,45^ (Fig. 9c). The PVRFZ has been suggested to contain contralateral callosal fibers in human^44^, which is in agreement with murine contralateral callosal fibers positioned above VZ/SVZ that we observed in Figs. 7f and 8i, j. In addition, we found ELAVL4-protein to be expressed in ventral RG after 17 PCW, at later stages of human neurogenesis when CELF1 expression decreases. We found CELF1 to be enriched in ventral RG in the VZ during 10 and 13 PCW and decreased by 17 and 20 PCW (Fig. 9c). Thus, CELF1 had a contrasting temporal expression pattern to ELAVL4 in the VZ (Fig. 9c), similar to what we observed in mouse neocortex (Fig. 3d). In addition to confirming the relevance of our findings for human neocortical development, our findings identify markers of early and late human ventral RG.

*ELAVL4* mRNA expression was present in the VZ at all stages (Fig. 9d), including early on when ELAVL4 protein is low (Fig. 9c). The discrepancy between *ELAVL4* mRNA and ELAVL4 protein expression is similar to what we observe in mouse early RG (Figs. 1–3). These findings demonstrate conserved expression patterns and suggest at least partially conserved ELAVL4/CELF1 regulatory mechanism between early and late stages of neurogenesis in both mouse and human.

### Multiple mRNAs associated with neurodevelopmental disorders are translationally regulated in developing neocortices

Given the conservation of the expression patterns of ELAVL4 and CELF1 in ventral RG during neocortical development in humans, we sought to explore the disease implications of the translational regulatory mechanisms. Initially, evolutionary constraint scores were computed by the Exome Aggregation Consortium (ExAC) as a measure of intolerance to damaging mutations^46^. We found that RBPs are highly constrained, including the members of the ELAVL family (Supplementary Fig. 10). Next, we performed enrichment analyses for published mRNA targets of the neuronal ELAVLs (ELAVL1, CELF1, and CELF4). Various degrees of enrichment were found in risk genes for neurodevelopmental disorders (NDDs) as defined by the DDG2P database, intellectual disability (ID; in OMIM), epileptic encephalopathy (EE; in OMIM) and ASD^47^ (Fig. 9e, Supplementary Fig. 10a). We also found several translationally regulated mRNAs to be targets of an RBP associated with ID and ASD, Fragile X mental retardation protein (FMRP)^48,49^ (Supplementary Data 3). The human orthologues of the translationally-regulated mRNAs during neocortical development (Fig. 1, Supplementary Data 1) were also found to be constrained (Supplementary Fig. 10b).

Given that these genes are under strong selective pressure, we conjectured that they might be essential for physiological neurodevelopment and vulnerable targets in NDDs. To explore this hypothesis, we cross referenced the list of differentially translated mRNAs at E16 with risk genes for NDDs in the DDG2P database; 61 of the 850 unique human orthologs derepressed at E16 are NDD genes (57 OMIM disease genes); 62 of the 1,070 genes that are repressed at E16 were NDD genes (59 in OMIM) (Supplementary Data 2). The mouse orthologs of genes associated with EE (e.g., *GRIN1* and *GABRA1*), ID (e.g., *MEF2C*), and ASD (e.g., *CHD8*, *MYT1L*), often in co-morbidity, were amongst mRNAs showing robust change in their polysome association (Fig. 9f; Supplementary Data 2). Collectively, these findings indicate that genes subjected to translational control during neocorticogenesis, including RBPs, are under strong selective pressure and might contribute to the vulnerability leading to NDDs.

## Discussion

**Fig. 10:**
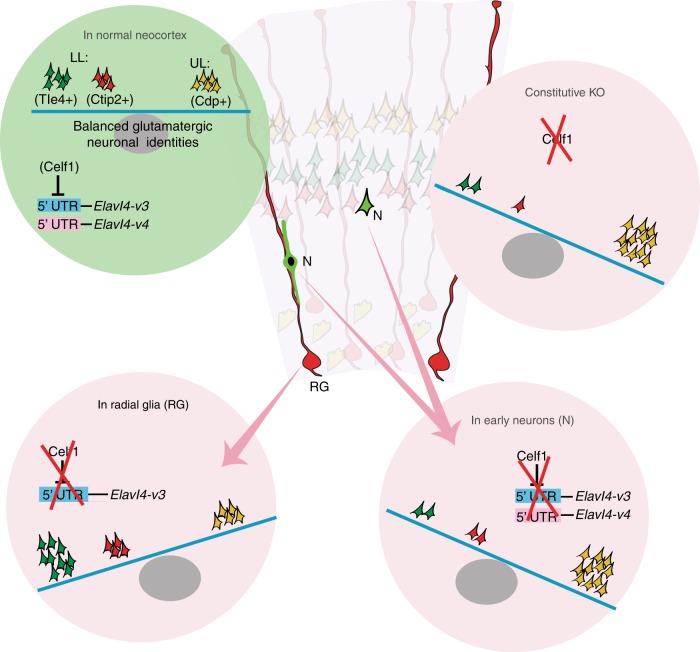
Summary graphic. Development of glutamatergic neurons is regulated by Celf1 translational repression of *Elavl4* through its 5ʼ UTRs.

The data contained herein demonstrate that one mechanism to achieve the incredible diversity of glutamatergic neurons is translational control by specific RBPs acting on alternative 5′ UTRs of corresponding mRNAs. We demonstrate that identity, subpopulation balance, and connectivity of neocortical glutamatergic neurons are dependent on translational control of *Elavl4* isoforms at the 5′ UTRs by Celf1 (Fig. 10). Previously, selective isoform-specific protein synthesis (mRNA translation) and the role of timed releases of translational breaks in any system was poorly understood.

To our knowledge, our study is the first genome-wide report of isoforms that are translationally repressed and derepressed during neocortical neurogenesis; many of these translationally-regulated genes turn out to be implicated in the etiology of NDDs. Our findings reveal that a robust set of mRNAs and their isoforms are poised to be translated rapidly at an appropriate time point. This sets up not only a highly plastic environment, but also a vulnerable one during cellular development.

The pattern of timed translational repression/derepression we revealed applies to *Elavl4* isoforms. The diverse effects of translational control on *Elavl4* isoforms within early and late RG and neurons is due to their alternative 5′ UTRs. The presence of specific 5′ UTRs in *Elavl4* isoforms reversed LL subpopulation-specific glutamatergic phenotypes that were seen with the OE of unregulated *Elavl4*. The differential identity acquisition seen in our cell subtype-specific experiments may further rely on available cell-specific transcripts that RBPs selectively act upon; as 5′ UTR-driven expression favors expression in one subtype identity over another. *Elavl4* isoforms also teach us that the significance of an mRNA cannot be guessed by its abundance. While *Elavl4-v3* and *Elavl4-v1&v4* are heavily regulated by Celf1, *Elavl4-v2* is less influenced by Celf1. Yet, *Elavl4-v2* is the isoform with the highest abundance. Besides differences in their 5′ UTRs and expression patterns, *Elavl4-v3* and *-v4* exhibit important differences in their “linker” regions positioned between the RNA binding domains, which was found to determine its association with polysomes^50,51^. Therefore, both the UTR and CDS may account for the differential phenotypes that are reflected in the isoform-specific experiments of our study.

Our screen led us to investigate another RBP, Celf1, which acts as an upstream regulator of *Elavl4* at its alternative 5′ UTRs. We found that Celf1 suppressed translation of distinct *Elavl4* isoforms by binding their alternative 5′ UTRs. Mutation of Celf1 binding sites also abolished the subpopulation phenotype observed upon OE of *Elavl4* isoforms with their native 5′ UTRs. Given the number of common binding motifs associated with other RBPs in our screen, Celf1 is likely only one example of an RBP that is an isoform-specific translation regulator and that has broad impacts across cell types during neocortical neurogenesis. Nevertheless, our findings underscore the potential diversity in expression that alternative splicing and precise dynamics of selective isoform translation provides.

We also report for the first time that Celf1 silencing itself disrupted the identity, subpopulation balance, and connectivity of glutamatergic neurons. This phenotype can be reversed by silencing of the translationally derepressed *Elavl4* isoform, *Elavl4-v3*. The OE of Celf1 also leads to disrupted development of glutamatergic neurons that can be corrected by overexpressing *Elavl4-v3* bearing its native 5′ UTR. These findings reaffirm that *Elavl4* is downstream to Celf1 and demonstrate that both an upstream regulator and its mRNA target with native regulatory sites work together to give us a balanced neocortex. It is reasonable to expect that other regulators like miRNAs or other RBPs will also compete with Celf1 to not only bind, but also to regulate Elavl4 expression^20,52^.

There is immense potential for increased understanding of NDDs by studying the mechanisms that control translation during development. Our study has revealed the magnitude of translationally repressed and derepressed mRNAs and their associated isoforms. Disrupted functions and mutations of RBPs are linked with several NDDs associated with abnormal neocortical development. This is particularly interesting given the various neuroanatomical aberrations caused by Celf1 and Elavl4 disruptions. Indeed, prenatal deletion of *Elavl4* has been associated with disrupted development of neocortical circuits, misbalance of glutamatergic identities, seizure susceptibility, and autism-like repetitive behaviors^9,19,51,53,54^. Another paradigmatic example of an RBP associated with NDD is Fragile X syndrome, which is caused by loss of the FMRP and the translational control it executes^49,55–57^. FMRP^49,58^, ELAVL1^13^, neuronal ELAV, Celf1 and Celf4^59^ (Fig. 9e), Celf6^60^ and CPEB4^61^ target mRNAs are encoded by ASD risk genes^62–64^. We found that both CELF1 and ELAVL4 are expressed in developing prenatal human neocortices suggesting their roles and vulnerability to disruption during development. RBPs can thus have diverse effects in different NDDs by causing global misregulation of their mRNAs. This suggests a previously overlooked mechanism contributing to the manifestation of comorbid conditions. Future studies are needed to elucidate the complete picture of collaborative and competitive differences in the control of isoform-specific 5′ UTRs, which we can now appreciate as critical in fully understanding how neocortical RG and neurons develop.

## Methods

### Animals

Experiments involving animals were carried out in accordance with Rutgers University Medical School’s Institutional Animal Care and Use Committee. For timed pregnancies, adult pregnant female CD-1 mice were purchased from Charles River Laboratories. The day of vaginal plug discovery was considered embryonic day 0.5 (E0.5). The *Elavl4-GFP* transgenic line was obtained from GENSAT (www.gensat.org) and previously reported^5^. *Elavl4* KO, *Celf1* KO, *Nestin-GFP*, and *Tbr2-GFP* transgenic mice were described previously^9,28,30–33,36^. For all embryonic experiments, mice of either gender were used and gender was not determined. Gender was also not determined in human fetal tissue.

### Postmortem human brain tissue

All experiments on human tissue were carried out in accordance to protocols approved by the Institutional Review Board (IRB) of the Ethical Committee of the School of Medicine, University of Zagreb and were carried out in accordance with the Declaration of Helsinki 2000. Fetal brain specimens are part of the Zagreb Brain Collection, received from clinical hospitals affiliated with the School of Medicine, University of Zagreb^45^. Tissue was fixed with a post-mortem delay of less than 6 h. Post-conceptual weeks (PCW) were determined according to the clinical pregnancy records and crown-rump length.

### Polysome fractionation

Polysome fractionation of neocortices was performed as previously described^10^. Briefly, embryonic neocortices were dissected in ice-cold HBSS media and immediately frozen on dry ice (*n* = 6 brains per spin). Tissue was homogenized in polysome extraction buffer (PEB) and the input was layered on top of a 10–50% sucrose density gradient in thin wall polypropylene centrifuge tubes (Cat. no. 347357), with surplus input being stored for further processing. Gradients were spun on Sorvall Discovery M120SE in Sorvall S55S rotor with swinging buckets (#18507) at 39,000 RPM for 50 min. Fractions were collected using Fluorinert FC-40 (Cat. no. F9755) and a Brandel fraction collector (no. 621140007), with UV absorbance recorded at 254 nm (Brandel UA-6). Samples were stored at −80 °C until further processing.

N2a cell cultures were grown in 100 mm plates to 75-85% confluency and transfected with 15 µg of plasmid using Lipofectamine 2000 in a 2:1 ratio (Lipofectamine:DNA). A 15 min cyclohexamide treatment (0.1 mg/ml) was conducted at the end of the 48-hour transfection. Polysome fractionation of frozen neocortices and cell pellets started with lysis in PEB, freshly supplemented with EDTA-free protease inhibitor (Santa Cruz Biotechnology; no. sc-29131), RNaseOut (Invitrogen; no. 100000840), 20 mM DTT (Invitrogen; no. NP0009), and 0.1 mg/mL cyclohexamide (Santa Cruz Biotechnology; no. sc-3508A). Lysis was conducted on ice for 10 minutes, pipetting up and down every two minutes. Lysate was spun for 10 min at 4 °C and 5 K RPM; the resultant supernatant was spun for 5 min at 4 °C and 14k RPM. Samples were measured using a Nanodrop under the A_280_ wavelength to normalize loading to total protein content. Sample was loaded onto a 10-50% sucrose gradient. Gradients were prepared the evening before in 2 ml polyallomer tubes (Beckman Coulter; no. 347357) and left to stabilize to a continuous gradient overnight at 4 °C. Gradients were spun for 120 min using a Thermo Fisher Sorvall MX 120+ micro-ultracentrifuge and the Sorvall S-55-S swinging bucket rotor at 4 °C. Fractions were collected until the presence of Fluorinert FC-40 (Sigma; no. F9755) was detected. Samples were stored at −80 °C until further processing.

RNA was isolated using TRIzol reagent and mostly according to the manufacturer’s protocol briefly: the nucleic acid from each fraction was isolated using a 3:1 ratio of TRIzol:sample. The mixture was washed with chloroform twice to isolate and purify the aqueous phase. Total RNA was precipitated with one volume of isopropanol/glycogen (Thermo Fisher; no. R0551). The pellet was washed in 75% ethanol and the resulting pellet was dried and resolubilized with Molecular Biology Grade water (Corning; no. 46-000-CV) supplemented with RNAseOut for ~30 min, vortexing frequently. DNase treatment was conducted according to the manufacturer’s protocol (Invitrogen; no. AM1907); DNase was inactivated with phenol:chloroform (Fisher Scientific; no. BP1754I). RNA was further purified by chloroform extraction and precipitated with 3 M sodium acetate (G-Biosciences, no. R010). Samples were stored at −80 °C in 100% ethanol until further processing.

qRTs were conducted as described in the relevant methods subsection. Polysome inputs were diluted 1:25 for *Elavl4-v1/4* and *Elavl4-v2* assays and normalized to *Gapdh*. Fractions were diluted 1:5 for *Elavl4-v1/4* and *Elavl4-v2*. *Elavl4-v3* assays were run with undiluted sample. Non-template controls were used to determine background levels of amplification and verify fraction amplification. Polysome fractionation was recorded with analog and digital traces recorded by UV-vis at 260 nm and rRNA *18 S* was tested for in each fraction. CELF1 short OE and KD were confirmed by qRT using a *Celf1-pan* TaqMan probe. Ct values were used in comparison analysis. We first applied the closure operator so that the components added to 1.0^65^. Differences in polysome fractions (e.g., HP - F) were tested between conditions using Welch’s *t*-test. Nominal significance levels are indicated in the figures.

### RNA-seq, GO, and KEGG analysis

RNA-seq was performed at RUCDR Infinite Biologics™ using Illumina technology and analyzed as described previously^10^. Briefly, RNA was isolated from input and polysome fractions by using TRIzol LS (Life Technologies; no. 10296028) following the manufacturer’s protocol. Next, equal volumes of RNA extracted from fractions 4–7 were pooled together for analysis of “40S–60S–80S-associated cytoplasmic RNA” and equal volumes of RNA extracted from fractions 9–12 were pooled together for “polysome-associated cytoplasmic RNA”. Three biological replicates were analyzed for input, 40S–60S–80S, and polysome RNA at E13 and E16 in WT (18 total samples). Sequencing libraries were prepared by using the Illumina TruSeq RNA Sample Preparation Kit v2 according to the manufacturer’s protocol. Libraries were quantified by using the Library Quantification Kit Illumina/Universal (KAPA Biosystems) and then diluted and symmetrically pooled. We performed 2 × 75-bp paired-end sequencing using the Illumina Hiseq2500. Sequencing data have been deposited in the Gene Expression Omnibus (GEO) database, www.ncbi.nlm.nih.gov/geo (accession no. GSE77674).

Results were aligned with the mm10 mouse genome found in the University of California Santa Cruz transcript map (Illumina iGenomes) using TopHat (v 2.1.1), and comparisons between groups were made in Cufflinks (v 2.2.1) and cummerbund (v 2.22.0)^66^. Significant differences were judged using a 5% false discovery rate (FDR). The detection of up- or down-regulated mRNAs is more sensitive to lower expression levels in the polysome fraction than in total cellular RNA, so differences in polysome mRNAs were assessed whether these mRNAs were detected in total or not. Relatively few translationally derepressed mRNAs were below the detection threshold in total RNA (97 out of 971 mRNAs). Lists of regulated genes were assessed for enrichment of functional groups or pathways using g:Profiler^26^. Transcript isoforms that were significantly increased or decreased (from E13 to E16) in polysomes, but unchanged in total RNA were used to prepare unique Ensembl Gene ID lists (using the biomaRt package in R)^67^ for upload to the g:Profiler site. Significantly enriched gene ontology groups, as represented by the best term per parent ontology chosen by p-value, are shown (Supplementary Fig. 1). Similarly, the best terms from a KEGG pathway search are included.

### Identifying common RNA-binding motifs

Transcript isoforms that were significantly up- or down-regulated in E13 vs. E16 polysomes and unregulated in total RNA were selected and used to download FASTA-formatted cDNA sequences using biomaRt. For comparison, all known cDNA FASTA sequences were downloaded. Motifs enriched in either the up- or down-regulated polysome lists compared with all cDNAs were identified using Homer v 4.9^68^, with the “-rna” flag.

### RNA-binding proteins

RNA-binding proteins (RBPs) were selected from the census curated by Gerstberger et al.^69^ filtered for RBPs with mRNA as consensus RNA target and “established” as supporting evidence as (205 unique genes).

### Primary antibodies

The following primary antibodies and dilutions were used: mouse anti-Elavl4 (anti-HuD; Santa Cruz Biotechnology (SCB), 1:1,000, sc-28299), rabbit anti-Pax6 (Biolegend previously Covance, 1:250, 901301 previously PRB-278P), rabbit anti-Tbr2 (Abcam, 1:250, ab23345), rabbit anti-Cdp (SCB, 1:250, sc13024), rat anti-Bcl11b (SCB, 1:250, sc-98514), mouse anti-Celf1 (SCB, 1:100, sc20003), rat anti-Ctip2 (Abcam, 1:250, ab18465), mouse anti-Tle4 (SCB, 1:250, sc-365406), chicken anti-GFP (Aves, 1:1000, GFP-1020), mouse anti-Gapdh (Millipore Sigma, 1:2000, MAB374), mouse anti-Satb2 (Abcam, 1:250, ab51502), rabbit anti-Nestin (Sigma Aldrich, 1:100, N5413), chicken anti-N-Cadherin (Takara, 1:250, M110 Clone NCD-2), rabbit anti-NeuN (Millipore Sigma, 1:500, ABN78), rabbit anti-pH3 (Millipore Sigma, 1:1000, 06-570), rat anti-Brdu/Cldu (Novus, NB500-169), mouse anti-Brdu/IdU (BD Biosciences, 1:100, B44), and rabbit anti-Renilla (Thermo Fisher, 1:1000, PA1-180). Appropriate species-specific Donkey secondary antibodies were used at a 1:250 dilution and were obtained from Jackson ImmunoResearch.

### Plasmids

Elavl4 isoform specific overexpression plasmids were commercially obtained from Genecopoeia: *Elavl4-v1* (Prod ID: EX-Mm30154-M46), *Elavl4-v2* (Prod ID: EX-Mm26736-M46), *Elavl4-v3* (Prod ID: EX-Mm29523-M46), and *Elavl4-v4* (Prod ID: EX-Mm29524-M46). *Celf1* isoform specific overexpression constructs were obtained from Origene: *Celf1 Long* (*Variant 1*; Cat# MC217278) and *Celf1 Short* (*Variant 2*; Cat# MC216621). *Celf1* shRNAs were commercially obtained from Origene; *Celf1* mouse shRNA Plasmid (Locus ID 13046) (Cat# TF514753B), and Genecopoeia; *Celf1 Variant 1 Long* (Prod ID; MSH031592-mU6) and *Celf1 Variant 2 Short* (Prod ID: MSH043146-mU6). *Elavl4-v3* shRNA was custom made (Origene, Cat# HC121119A, TTTAAGAGAAGAGTCGAAGCGCTGCGAGA).

The pCdk5r-Fezf2-IRES-GFP plasmid was generously shared as a gift from Paula Arlotta. Briefly, to clone the *Elavl4-v3* (Prod ID: EX-Mm29523-M46) and *Elavl4-v4* (Prod ID: EX-Mm29524-M46) overexpression under the Cdk5r promoter, the Hpa1 and Xho1 were used to digest the pCdk5r vector and the In-Fusion HD Cloning Plus kit (Clontech, Cat. no. 638909) was used to insert *Elavl4-v3* or *-v4* clone. The new construct lacks the original cloning sites. The pNestin-EGFP plasmid was obtained from Addgene (# 38777). The *Elavl4-v3* and *Elavl4-v4* plasmids above were digested by Sac1 and Spe1 to remove the CMV promoter, and the In-Fusion HD Cloning Plus kit was used to insert the Nestin promoter of pNestin-EGFP plasmid into *Elavl4-v3* and *Elavl4-v4* vectors. Clones were confirmed through sequencing.

### *Elavl4* UTR reporter constructs

The full-length 5′ UTRs of *Elavl4* variants were amplified from wild type mouse genomic DNA. The *Elavl4-v3* 5′ UTR sequence (1–97 bp) was amplified with the forward primer 5′-AAAAActtaagGCGCGGGACCCAGTGAGAA-3′ and the reverse primer 5′- AAAAAccatggCTTCGCGGAGGCGGGGT-3′. The PCR products were digested with AfIII and NcoI and ligated upstream of the Renilla gene in the LightSwitch 5′UTR reporter vector (SwitchGear Genomics, #S690005). Four *Elavl4-v4* 5′ UTR subclones (1–341, 1–143, 144–341, and 144–248 bp) were similarly amplified and cloned. All primer sequences can be found in Supplementary Data 4. All other *Elavl4-v3* and *Elavl4-v4* 5′ UTR subclones were synthesized by IDT with sticky ends of the above-mentioned restriction sites for direct ligation. The *Elavl4* 3′ UTR was amplified with Q5 High-Fidelity DNA Polymerase (NEB) and cloned into the XhoI and NotI sites of psiCheck-2 vector (Promega), downstream of the Renilla luciferase gene. All UTR clones were confirmed with sequencing.

Translationally-relevant binding sites in *Elavl4* 5′UTRs were determined by translation assays using subclones described above. These 12 bp sites in the *Elavl4-v3* and *Elavl4-v4* 5′UTRs were mutated by changing the WT bases to their complements. For reporter gene constructs, full length *Elavl4* 5′UTRs with binding site mutated (BSmut) were synthesized by IDT bearing AfIII and NcoI sites at the two ends, and then subcloned upstream of the Renilla CDS in the pLightswitch vector above. The same strategy was used for overexpression constructs, except that the synthesized 5′ UTR fragment contains flanking sequence from CMV-*Elavl4*-OE CDS vectors (Origene) for subsequent subcloning to SacI and Bsu36I sites. All clones were confirmed by sequencing.

### *Elavl4* isoform diagram

The mouse *Elavl4* isoform diagrams were retrieved from AceView.

### Celf1 isoforms protein alignment

Celf1L and Celf1S protein sequences were obtained from USCS genome browser. Protein sequences were aligned using Clustal Omega.

### Immunohistochemistry on mouse brain tissue

Standard methods were used for IHC as described previously^10^. Briefly, embryonic brains and brains from postnatal day 0 (P0) were dissected and fixed by immersion for 6 to 12 h in 4% paraformaldehyde (PFA, Sigma-Aldrich, Cat. no. 158127) in phosphate buffered saline (PBS, Corning cellgro, Cat. no. 21-040-CV), pH 7.4, as described in Ref. ^10^ (Embryonic brains for 6–8 h, P0 brains for 10–12 h). After fixation, the brains were washed three times in 1× PBS and stored in 30% sucrose for later use. Brains were coronally-sectioned at 70–80 µm using a Leica VT1000S vibratome. Free-floating sections were then incubated in blocking solution (normal donkey serum (Jackson ImmunoResearch, Cat. no. 017-000-121), albumin (Biomatik, Cat. no. A2134), 0.2% Glycine (BDH, Cat. no. BDH4156), 0.2% l-lysine (Sigma, Cat. no. L5501), and 0.4%Triton (Sigma, X-100)) gently shaking for 2–4 h at room temperature. Following incubation in blocking solution, the sections were immediately transferred to a solution of primary antibody resuspended in blocking solution +0.4%Triton X-100 and incubated, gently rocking, overnight at 4 °C. The following day, sections were washed 3 times in 1x PBS before being transferred to a secondary antibody solution diluted in blocking solution without Triton X-100 for 1–2 h gently shaking at room temperature. Next, sections were washed again for three times in 1x PBS before being incubated in 1 μg/ml of DAPI (Fisher Scientific, Cat. no. D1306) for 10 min. Finally, sections were washed two more times in 1x PBS before being mounted with Vectashield mounting media (Vector Laboratories, H1000).

### Immunohistochemistry on human brain tissue

Tissue was fixed in 4% PFA (Biognost, cat. no. FNB4) for up to 48 h, dissected coronally in three blocks, embedded in paraffin (Merck, cat. no. 107300) and sectioned on a microtome (Leica, SM2000R) at 20 μm thick sections. Prior to immunohistochemistry, a standard process of deparaffinization was performed in a series of xylol and alcohol. After four washes in 1x PBS, blocking solution containing 1% BSA and 0.5% Triton X-100 in PBS was applied on sections for 1 h. Blocking solution was replaced with primary antibodies (mouse anti-Elavl4, 1:1000; rabbit anti-Pax6, mouse anti-Celf1, 1:200) diluted in blocking solutions and kept overnight at 4 °C. After incubation, sections were 3× washed in 1× PBS and appropriate secondary antibodies (donkey Alexa Fluor 546 (cat. no. A10036) and Alexa Fluor 488 (cat. no. A21206), diluted 1:1000, Thermo Fisher Scientific) were applied for 2 h. Following 3× washes in 1× PBS, DAPI (Sigma Aldrich, St. Louis, USA) was applied for 1 min according to manufacturer instructions. Sections were covered in Aqueous Mounting Medium (DAKO, Carpinteria, CA, USA).

### FISH on mouse brain tissue

IUE of CAG-GFP and either *5*′*UTR*-*Elavl4-v3* or *5‘ UTR-Elavl4-v4* under *Cdk5r* or *Nestin* promoters was done at E13. Brains were fixed after 24 (*Nestin* driven constructs) or 36 (*Cdk5r* driven constructs) hours with RNAse-free 4% PFA for 8 h. From the transfected brains, 70 μm thick sections were permeabilized with 500 μl of PBS-T (1X PBS with 0.5% Triton X-100) in a 24-well plate for 10 min at RT. After a rinse with 1X PBS, the slices were rehydrated in a solution of 10% formamide + 2X SSC for 10 min at RT with 2 ng/μl of a DNA probe (5′UTR-specific 90nt-cy3 or cy5, IDT). Sections were then hybridized in 250 μl of hybridization solution (50% formamide, 5X SSC, 5X Denhardt’s solution, 500 ng/μl Salmon Sperm DNA, 250 ng/μl Yeast tRNA) overnight at 37 °C using the HB-100 Hybridizer (UVP Laboratory Products). After two washes for 30 min at 30 °C using 10% formamide + 2X SSC, sections were stained 10 min at room temperature with DAPI and mounted^70^.

### In situ hybridization on human brain tissue

Fixed and paraffin-embedded coronal sections were mounted on slides, rinsed in 2 × SSC, prehybridized in hybridization buffer (HB) at 45 °C in hybridization oven for 1 h. Hybridization was performed in 100 ng/ml of digoxigenin-labeled anti-sense *hElavl4*-pan-DIG (IDT) or sense probes in HB, at 45 °C overnight. Following hybridization, the sections were washed in 2 × SSC, 2 × SSC/50% formamide, 0.1 × SSC/50% formamide and 0.1 × SSC, at 55 °C. DIG-labeled signal detection was performed with anti-DIG-AP antibody, as recommended by the manufacturer (Roche). Colorimetric detection was done using nitroblue tetrazolium (NBT) and 5-bromo-4-chloro-3-indolylphosphate (BCIP) color developing substrate (Promega). Color development reaction was stopped by transferring sections into 10 mM Tris–HCl, pH 8.0, and finally in 1 mM EDTA. Sections were analyzed and images taken with high-resolution slide scanner NanoZoomer 2.0RS (Hamamatsu).

### Neurogenesis analysis

Animals were bred for timed pregnancies to E13. In addition to standard handling of embryonic tissue, intraperitoneal injections were administered to pregnant dams to allow for thymidine analog incorporation. Cldu (Sigma; no. C6891), dissolved in 1 × PBS, was injected (50 mg/kg) at E12, 24 h prior to extraction of pups. Idu (Sigma; no. I7125), dissolved in 7 mM NaOH in 1 × PBS and made fresh daily, was injected (50 mg/kg) 1 h before brain fixation. Once brain sections were cut at 70 μm thickness, to allow for Cldu/Idu antibody penetration, antigen retrieval was performed. Sections were treated with 1 M HCl for 15 min shaking at room temperature, followed by a 15 min stationary treatment with 2 M HCl. Acid was washed off with 1 × PBS, 4 washes 5 min each. Our standard IHC protocol was followed thereafter. Cortex was imaged with a 20x objective. Files were opened in Fiji (NIH; ImageJ ver. 2.0.0-rc-43/1.51r) and all cells in cortex were marked using the Cell Counter plugin (license GPLv3). Cells were counted if they were between the pial surface and the VZ, and if they were positive for DAPI staining. Positive staining in individual channels was determined first. Then colocalizations were determined by the presence of two different markers on the same cell. Two-tailed unpaired t-tests were used to test comparisons for significant differences.

### Confocal imaging

All images except from human were taken with an Olympus BX61WI confocal microscope using 10 ×, 20×, or 60× objectives and processed using Fluoview FV-1000. Human IHC was analyzed using Leica TCS SP8 X FLIM confocal microscope. All representative images and images used in analysis were taken with the same confocal settings per experiment to allow for accurate comparisons of fluorescent intensity. Sections were imaged from the top of the cortical plate to the VZ and merged in the photo editing software Gimp2 and Fiji^71^. For quantifications of binned images, a rectangle approximately a third of the width of the image was drawn from CC to the tops of the apical tufts at the pia. The rectangle with split into ten equal bins from CC to pia. Cells were counted per condition as appropriate for the experiment, either GFP+ or GFP+ colocalized with a layer marker. DAPI was used to confirm cell nuclei.

### RNA immunoprecipitation

Six of E13 or E16 neocortices were dissected, pooled together, and considered to be one biological sample. Three biological samples were used for RNA immunoprecipitation (RIP) experiments. CELF1 RNA immunoprecipitation validated antibody (Millipore, Cat. no. 03-104) was used coupled to EZ-Magna RIP RNA-Binding Protein Immunoprecipitation Kit (Millipore, Cat. no. 17-701) and qRT-PCR analysis. For each target tested, three qRT-PCR technical replicates were done from each immunoprecipitation of each biological sample. To examine Celf1 binding to *Elavl4* 5′ UTR, the *Elavl4-v3* (or *v4*) 5′ UTR-Renilla + *Celf1S* OE constructs (0.2 and 1 µg/12-well plate) were co-transfected into N2a cells, which were then incubated for 3 days. 10^7^ cells were used for one RIP experiment of IgG or Celf1 and followed by the Millipore RIP kit. The effect of Celf1 binding to *Elavl4-v3* (or *v4*) 5′ UTR was measured with a Renilla TaqMan probe. 3–4 replicates of qRT-PCR were performed for each RIP.

### Western blot

Neocortical tissue and N2a cells were lysed in Tissue Protein Extraction Reagent (Thermo Scientific, Cat. no. 78510) and protein concentrations were determined using the Pierce 660 nm reagent (Thermo Fisher, Cat. no. 22660) on a Nanodrop ND-1000 spectrophotomer. Protein samples were analyzed using the NuPAGE system (Life Technologies) per manufacturer’s instructions and with 4-12% Bis-Tris gels (Cat. no. NP0335/6) and transferred to nitrocellulose membranes (GVS Life Sciences, Cat. no. 1215471). The resulting membranes were blocked for 1 h at room temperature with 5% nonfat dried milk (VWR, Cat. no. M203) and 10% fetal bovine serum (FBS) (Gemini, 900-108) in PBS with 0.4% Triton-X-100 (PBST). Primary antibody was added and the blots were incubated overnight at 4 °C gently shaking, followed by three washes in PBST. Secondary antibody diluted in 10% FBS in PBST was then added for 1 h. Development of blots was performed with ChemiGlow West Chemiluminescence Substrate Kit (Proteinsimple, Cat. no. 60-12596-00-2) and visualized with Gbox (Syngene). Quantification of band intensity was performed with GeneTools (Syngene) and Fiji^71^. The experimental protein of interest was normalized to Gapdh levels on the same blot. At least three biological replicates were performed for all analyses and an ANOVA or Student’s *t*-test was used to compare averages, as appropriate. *p* < 0.05 was considered significant.

### Laser capture micro dissection

Whole brains were dissected from developing embryos at E13 or E16 in an RNAse-free environment and flash frozen. Brains were then sectioned into 60 µm slices on a cryostat at −20 °C and placed onto Molecular Machines & Industries (MMI) RNAse Free Membrane Slides (MMI AG, Cat. no. 50102). The sections were rinsed with nuclease-free water and immersed for 1 min in 95% ethanol. Dried slides were subjected to microdissection using an MMI SmartCut Plus microscope. Neocortical compartments were determined by the cytoarchitecture of cresyl violet-stained tissue. Brains underwent laser capture microdissection of the ventricular zone and cortical plate. RNA was harvested from compartmentalized tissue using the RNAqueous-micro kit LCM protocol (Ambion). RNA from at least three brains was analyzed per experiment (*n* = 3) using quantitative real-time PCR (qRT-PCR, see below). Significant changes between conditions were analyzed using the ΔΔCt method. A student’s t-test or ANOVA was performed to evaluate significant changes and *p* < 0.05 was considered significant.

### Preparation and FACS sorting of GFP+ cells

WT female CD1 mice were crossed with Nestin-GFP or Tbr2-GFP males for timed pregnancies. The morning of plug observation was considered to be E0.5. Embryos were taken at E13 or E16 and observed under a dissecting microscope for GFP expression. GFP+ neocortices were dissected, pooled together. and single cell suspension per pregnant animal was prepared as described^13^. GFP+ cells were then FACS-sorted at the local Rutgers University Facility, spun down, and immediately homogenized in TRIzol (Invitrogen) for further RNA and protein isolation. Average values for cells obtained (from three separate spins): E13 Nestin-GFP, 348 K cells; E16 Nestin-GFP, 1.73 M; E13 Tbr2-GFP, 128 K; E16 Tbr2-GFP, 525 K. Sample purity was determined through ROI determination as well as post-sort visual examination on a fluorescent microscope at the facility.

### Quantitative real-time PCR (qRT-PCR)

RNA was isolated using TRIzol (Invitrogen) per manufacturer instructions and as previously described^10^. After ethanol was removed from the precipitate, the mRNA pellet was resuspended in 20–30 µL of water. Residual DNA was removed by incubating the mRNA with Turbo DNAse in DNAse buffer, and then the DNAse was inactivated with the DNAse inactivation reagent (Invitrogen, AM1907). The mRNA concentration was measured using a Nanodrop ND-1000 spectrophotometer. For LCM samples, 10–25 ng of total RNA was used per reaction. For all other reactions, 50 ng per reaction was used. The Applied Biosystems StepOne Real-Time System and reagents (TaqMan RNA-to-Ct 1-Step Kit, Thermo Fisher, #4392938) were used to perform qRT-PCR. The results were analyzed using the ∆∆Ct method with *Gapdh* as a normalization control unless otherwise specified. For qRT-PCR of *Elavl4* variants, commercially available TaqMan probes (Thermo Fisher) were obtained.

### In utero electroporation

In utero electroporation (IUE) was performed at E12, E13, or E16 and analyzed at E13, E17, P0, or P7 as previously described^9^. Co-transfections accomplished with approximately 1 µL of mix containing 3.5–4 µg/µL of the control vector or the vector of interest along with 4 µg/µl CAG-GFP reporter in a 4:1 ratio (construct:reporter). For rescue experiments, 8 µg/µL of the shRNA and overexpression vectors were combined and injected to achieve the final concentration of the OE experiments (~3.5–4 µg of construct, ~800–900 ng of reporter, per µl). For each IUE, at least three transfected neocortices were used in experiments and this is indicated in figure legends with the “*n*” value. Two to five sections from each transfected per staining were used in quantifications.

### Cell culture/transfections

Neuroblastoma N2a cells (ATCC, Cat. no. CCL-131) were grown in Dulbecco’s modified eagle medium containing 10% FBS (Gemini, Cat. no. 900-108), 1% GlutaMAX (Gibco, 35050-061), 1% sodium pyruvate (Gibco, Cat. no. 11360-070), and 1% penicillin streptomycin (Corning, Cat. no. 30-001-CI). TrypLE Express (Gibco, Cat. no. 12604-021) dissociation reagent was used for regular maintenance. Lipofectamine 2000 (Invitrogen, Cat. no. 11668-019) was used to perform transfections per the manufacturer’s protocol.

### Renilla luciferase reporter assay

N2a cells were seeded in 12-well plates and co-transfected the next day with 0.2 µg of either of the *Elavl4* 5′ UTR-Renilla reporter vectors and 1 µg of either the Ctrl OE, *Celf1S* OE, or *Celf1L* OE vectors. Forty-eight hours after transfection, cells were trypsinized with TrypLE Express (Gibco, Cat. no. 12604-021) and divided equally into two fractions. One fraction of the cells was spun down and lysed at room temperature for 15 min with 250 µL of 1× Passive Lysis Buffer (Promega, in Ref# E1910 Kit). The other fraction of cells was used for RNA isolation using TRIzol (Invitrogen). All protein lysates were quantified with the Pierce 660 nm Protein Assay reagent (Thermo Fisher) on a NanoDrop spectrophotometer and normalized to the same concentration with lysis buffer for equal loading. To measure the Renilla luminescence (REN), 10 µL of normalized protein lysate was mixed with 50 µL of working solution made from the LightSwitch Luciferase Assay Reagent (SwitchGear Genomics, Cat. no. 32035). REN readings were immediately performed on a GloMax 20/20 luminometer (Promega). The levels of Renilla mRNA (*Ren*) with 50 ng of Turbo DNase (Invitrogen, Cat. no. AM2238) treated RNA fraction were quantified by qRT-PCR using the custom made Taqman probe Rensp (Thermo Fisher). The REN luminescence readings were then normalized to *Ren* mRNA levels for each sample. Each assay condition was performed with at least three biological replicates. ANOVA or Student t-test statistical comparisons of REN/*Ren* between experimental conditions were performed. *P* < 0.05 was considered significant. The same approach was used for experiments with *Celf1* knockdown except that cells were harvested 72 h after co-transfection of reporter plasmids with the control shRNA or *Celf1* shRNA plasmid. Similar experiments were also conducted for *Elavl4* 3′ UTR reporter gene assay, except that the Dual-Luciferase Reporter Assay kit (Promega) was used for determining the Firefly Luciferase luminescence.

### mRNA decay assay

N2a cells were seeded in 100 mm dishes and transfected the next day with 30 µg of either the Ctrl OE, *Celf1S* OE, or *Celf1L* OE plasmids. Twenty-four hours later the bulk transfectants were reseeded evenly into 35 mm dishes and rested overnight. The cells were then treated with Actinomycin D (Sigma, Cat. no. A9415) at a final concentration of 5 µg/mL and incubated for 15 min to stop transcription before starting the time point. Cells were then harvested at 0, 2, 4 and 6 h for RNA isolation and qRT-PCR analysis of mRNA decay. The same approach was used for experiments with Celf1 knockdown (where the control shRNA or *Celf1* shRNA plasmids were bulk transfected) except that cells were left in 35 mm dishes for 48 h after replating and before Actinomycin D treatment.

### Primary neuronal cultures

Alternating pups were co-electroporated with Ctrl shRNA and CAG-GFP or *Celf1* shRNA and CAG-RFP and primary neuronal cultures were performed as described^13^.

### Constraint scores

Constraint metrics were obtained from the Exome Aggregation Consortium (ExAC), Cambridge, MA (URL: http://exac.broadinstitute.org) [May 2016]. We used the missense Z-score, computed as deviation of observed number of missense variants from the expected number, as a measure of intolerance to missense variation^46^. We used the probability of loss-of-function intolerance (pLI) as a measure of intolerance to loss-of-function mutations^46^. For the partitioning of genes in pLI tranches, deviation from the distribution observed for all genes was evaluated using Kolgomorov-Smirnov test (ks.test, R).

### Enrichment analyses

We extracted the 1216 unique PAR-CLIP targets of ELAVL1 (Lebedeva et al., 2011^72^, Supplementary Data 2, genes described as “Conservative targets”, PMID:21723171), 924 unique top HITS-CLIP targets of nELAVLs (Scheckel et al., 2016^73^, Supplementary File 1, PMID: 26894958), 431 human orthologs of the top 490 iCLIP targets of CELF4 (Wagnon et al., 2012^74^, Supplementary Data 1, PMID:2320943), 1439 unique HITS-CLIP targets of CELF1 (Le Tonquèze et al.^75^, Supplementary Data 3, genes described as “inLETONQUEZE=1”, PMID:27222809). We also used 915 unique genes in the Developmental Disorders Genotype-Phenotype Database (DDG2P, https://decipher.sanger.ac.uk/info/ddg2p) with “brain” included in organ specificity (referred to as NDD_DDG2P), 61 unique gene associated with EE in the Online Mendelian Inheritance in Man (OMIM) database (EE_OMIM), 142 unique genes associated with ID in OMIM (ID_OMIM), and 102 unique genes associated with ASD (ASD_Satterstrom) (PMID: 31981491). For the enrichment analyses, we used ELAVL1, nELAVLs, CELF4 and CELF1 genes are input sets, the NDD_DDG2P, EE_OMIM, ID_OMIM, and ASD_Satterstrom as target sets, and 19,584 unique human genes as background set. For each comparison between the input and target sets, we first constructed the empirical distribution by sampling the same number of genes as in the input set from the background set 10,000 times. The P value was then computed by calculating the number of sampled gene lists that had at least as many overlapping genes with the target sets as the input set, divided by 10,000 iterations.

The 915 NDD_DDG2P genes were also crossed with the 850 human orthologs of the 917 unique mouse genes de-repressed at E16, and with the 1,070 human orthologs of the 1,093 unique mouse genes repressed at E16. Overlapping genes were further mapped to OMIM.

### Quantification

Cell counting was done in double blind fashion where neither the person imaging nor quantifying knew the experimental condition. The percentage of colocalization between GFP and the glutamatergic identity markers of interest was quantified in Fiji and is presented as the fraction of total GFP+ cells. For the cell migration analysis, 10 equal sized bins from either (1) the top of the cortical plate to bottom of the cortical plate or (2) from the top of the cortical plate to the bottom of the VZ were drawn for each experiment. Binning conditions were kept constant across an experiment and the number of GFP+ cells in each bin was counted to be presented as the fraction of total GFP+ cells.

### Statistical analysis

Graph bars represent mean and ±SEM or mean and ±SD as noted in figure legends. The number (*n*) of replicates for each experiment is also noted in figure legends. Appropriate statistical tests were used (either unpaired Student’s *t*-test or one way ANOVA Tukey’s post-hoc test for multiple comparisons), also noted in figure legends. Statistical significance is considered achieved when *p* < 0.05 and is reported as: *p < 0.05, **p < 0.01, ***p < 0.001, ****p < 0.0001.

### Reporting summary

Further information on research design is available in the Nature Research Reporting Summary linked to this article.

## Supplementary information

Supplementary Information

Description of Additional Supplementary Information

Supplementary Data 1

Supplementary Data 2

Supplementary Data 3

Supplementary Data 4

Reporting Summary

## Data Availability

The unprocessed sequencing data have been deposited in the NIH GEO with accession number: GSE77647. Processed data used for the analyses are included in Supplementary Data files 1–3. The source data underlying Figs. 1g–j, 2b, 3b, 3e, 4b, 4d, 4g, 4h, 5a–e, 6d, 7b–e, 8e–h and Supplementary Figs. 1a, 2b, c, 4c–e, 5a, 5c–e, 6a–h, 7b, c, 8c, 9b, c are provided as a Source Data file.

## Source Data

Source Data
