# Supplementary material for: Translational derepression of Elavl4 isoforms at their alternative 5′ UTRs determines neuronal development
Source: Nat Commun. 2020 Apr 3;11:1674. doi: 10.1038/s41467-020-15412-8 (PMC7125149; doi:10.1038/s41467-020-15412-8)
Supplement: Supplementary file 1 — Supplementary Information [file 41467_2020_15412_MOESM1_ESM.pdf]

**Supplementary Information**

# Translational derepression of *Elavl4* isoforms at their alternative 5’ UTRs determines neuronal development

Popovitchenko et al., 2020

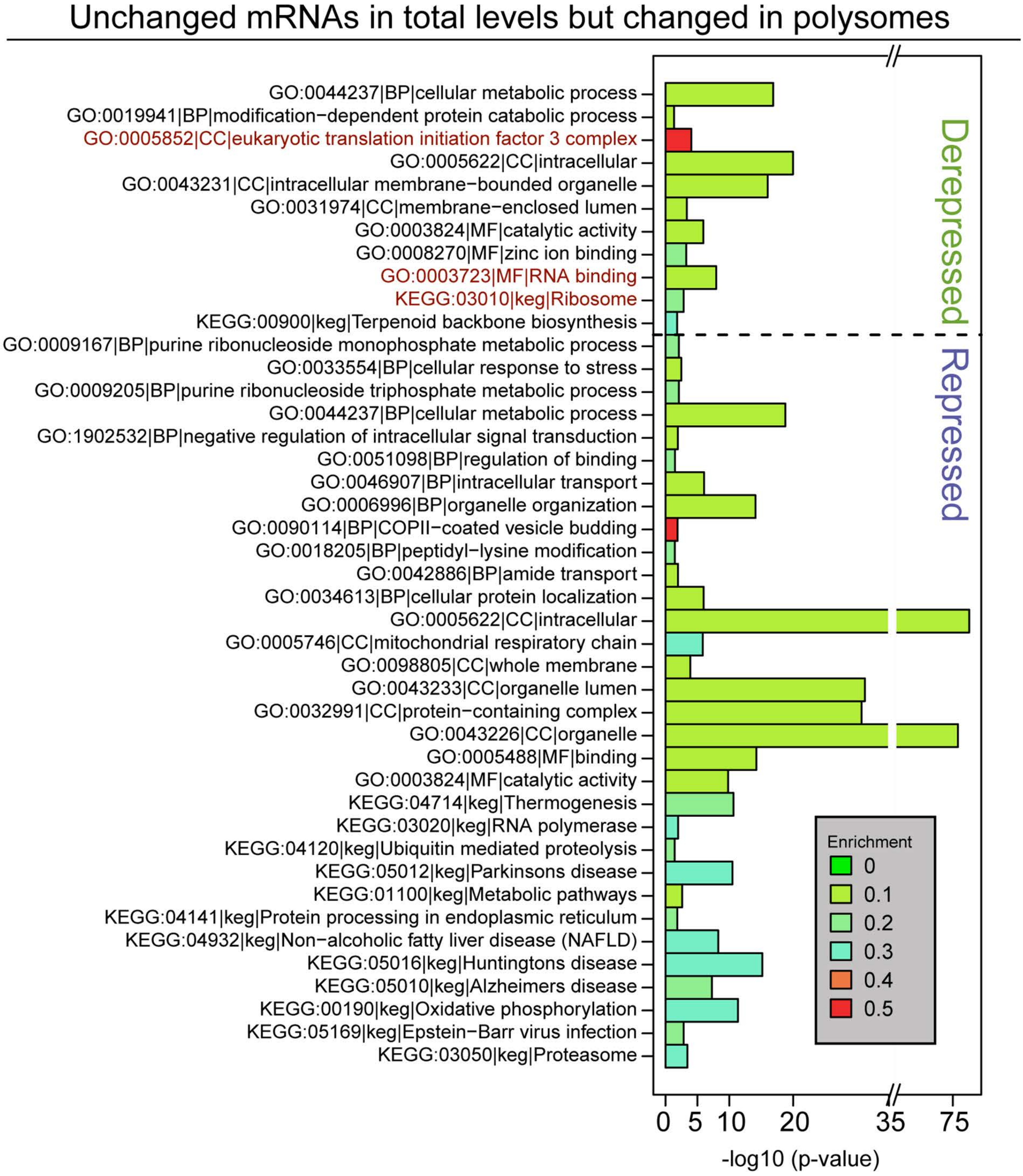


**Supplementary Figure 1. GO and KEGG pathway analysis using g:Profiler of isoforms unchanged in total levels but changed in polysomes.** Relative term enrichment (number of term-specific genes in the query list plus total term-specific genes divided by the total term-specific genes, or “Q+T/T” from g:Profiler) is represented by the color scale at right.

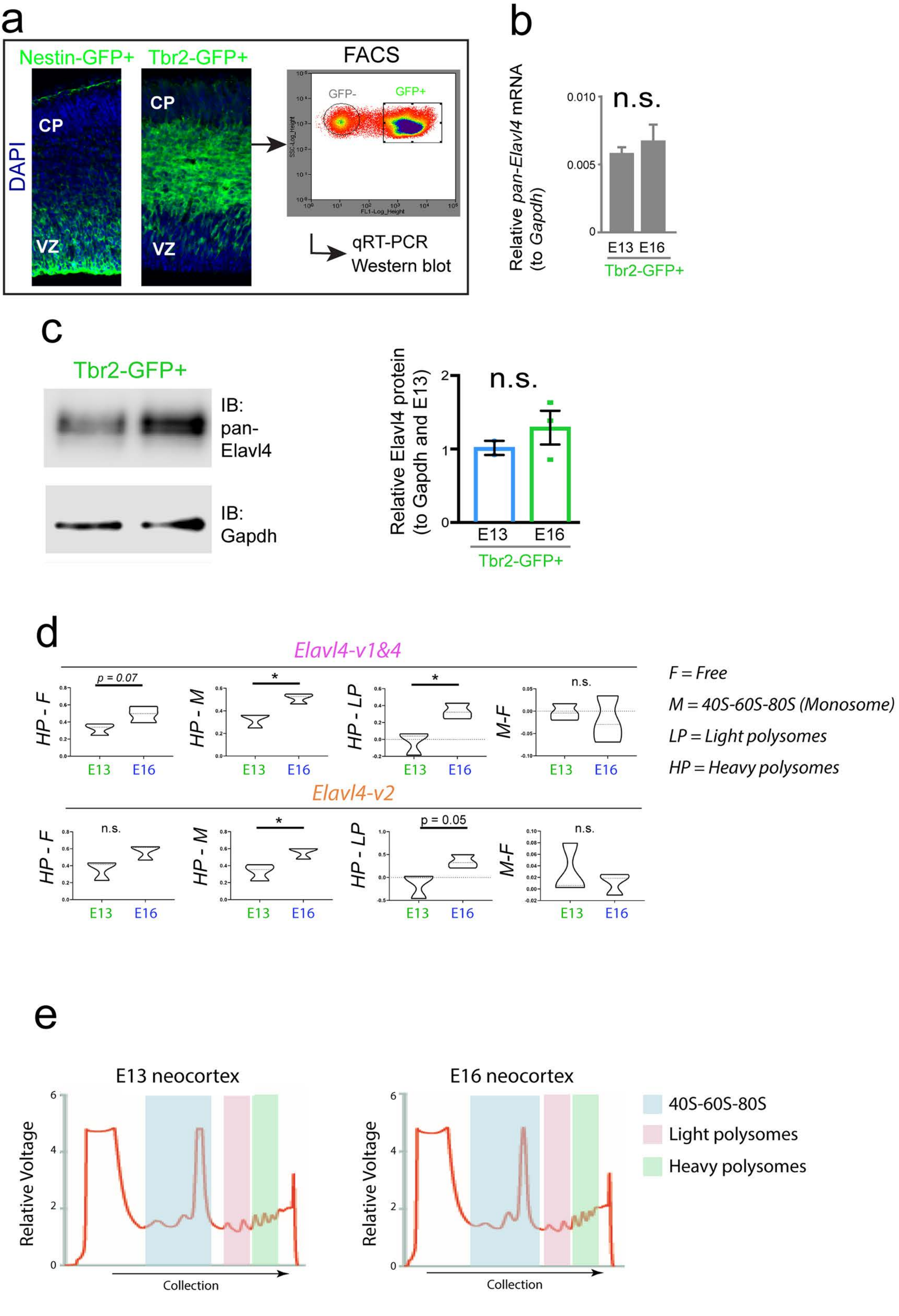


**Supplementary Figure 2. Elavl4 is translationally derepressed in developing prenatal neocortices.**

**a,** Schematic of the experimental design and workflow. Images of E16 transgenic Nestin-GFP+ and Tbr2-GFP+ mice used in this study (left). FACS sorted GFP+ cells were analyzed by qRT-PCR and Western blot.

**b,** Relative mRNA levels from FACS-sorted GFP+ cells determined by qRT-PCR (n = 3 FACS sorts). Data represent the mean and SEM. Data were normalized to *Gapdh*. Statistics: Student's t-test. *p < 0.05.

**c,** Western blot for Elavl4 on FACS sorted GFP+ cells from E13 (N=2 FACs sort) & E16 Tbr2-GFP (N=3 FACs sorts) neocortices quantified left . Gapdh was used as loading control. Data represent the mean and SEM. Data were normalized to Gapdh. Statistics: Welch's t-test. n.s. = not significant).

**d**, Polysome fractionation of E13 and E16 WT neocortices. Difference of each isoform between free (F), 40S-60S-80S monosome (M), light polysome (LP), and heavy polysome (HP) fractions is shown (n= 3 fractionations, 6 brains per E13 fractionation, 4 brains per E16 fractionation). Statistics: Welch's t-test. * p < 0.05, n.s. = not significant.

**e**, Representative polysome fractionation curves from E13 and E16 neocortices.

a

Common 5' UTR motifs in E16 translationally derepressed mRNAs

| Rank | Motif | P | log P | % of mRNA | RBP that binds to motif |
|---|---|---|---|---|---|
| 1 | CAUUCGCUGC | 1e-8 | -1.992e+01 | 9.54% | |
| 2 | UUGCUUUGCG | 1e-7 | -1.815e+01 | 2.13% | Elavl1/HuR |
| 3 | GCUGGCCCCGCC | 1e-7 | -1.758e+01 | 2.02% | Elavl4/HuD |
| 4 | ACCGCCAA | 1e-7 | -1.631e+01 | 18.86% | |
| 5 | AGUUCCGGGCCG | 1e-6 | -1.544e+01 | 3.03% | |
| 6 | GCGCAUGCGC | 1e-6 | -1.519e+01 | 6.06% | |
| 7 | AAGUGUAA | 1e-3 | -8.120e+00 | 7.30% | Elavl1/HuR |
| 8 | AAAAAUAAAA | 1e-2 | -6.713e+00 | 4.38% | Pabpc1, Ptbp1, Elavl1/HuR |
| 9 | UGUGUGUGUGUG | 1e-2 | -5.552e+00 | 4.71% | Celf1/Cugbp1, Celf4/Cugbp4 |

b

Common 5' UTR motifs in E16 translationally repressed mRNAs

| Rank | Motif | P | log P | % of mRNA | RBP that binds to motif |
|---|---|---|---|---|---|
| 1 | UCCGGAAGCG | 1e19 | -4.513e+01 | 8.99% | Psi |
| 2 | GCCGCCGCCGCG | 1e13 | -3.114e+01 | 90.74% | Ybx1 |
| 3 | GCGCAUGCGCAG | 1e12 | -2.953e+01 | 6.63% | Ptbp1 |
| 4 | GUAGGGCU | 1e9 | -2.232e+01 | 19.98% | Msi2, Hnrnpa1, Hndnpa2b1 |
| 5 | AGUCCGUGGC | 1e7 | -1.823e+01 | 7.81% | Ptbp1, Ptbp2 |
| 6 | GCCCGGCCGGCC | 1e7 | -1.771e+01 | 79.29% | Gtf3a |
| 7 | CUGAGGAGGCCG | 1e7 | -1.757e+01 | 57.31% | Sfrs1, Sfrs7 |
| 8 | CCGCCCGCGCCC | 1e6 | -1.577e+01 | 66.39% | Ptbp1, Ptbp2 |
| 9 | GAGCGGUUAG | 1e5 | -1.337e+01 | 1.09% | Ptbp1 |
| 10 | CUGCUGCUGCUG | 1e4 | -1.091e+01 | 53.68% | Ptbp1 |
| 11 | AGCCAGCCAGCC | 1e4 | -9.276e+00 | 0.73% | Zpf36 |
| 12 | AAAAAAAAAAAA | 1e2 | -6.833e+00 | 1.27% | Pabpc1, Ptbp1, Elavl1 |
| 13 | AGAAGGCAAGGA | 1e1 | -3.508e+00 | 0.45% | Sfrs1 |

c

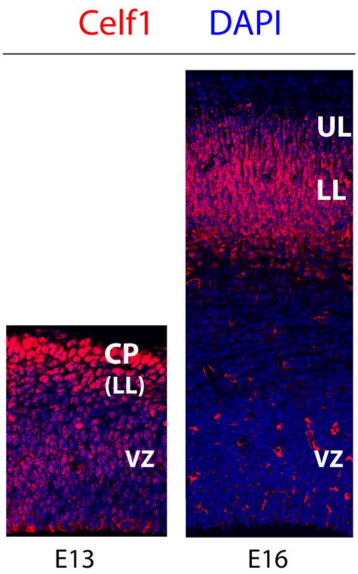


d

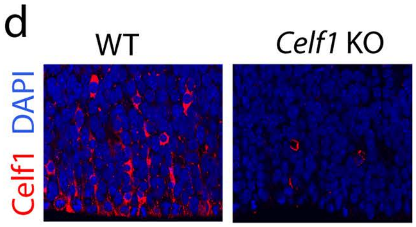


**Supplementary Figure 3. Binding motifs in 5' UTR of E16 derepressed and repressed mRNAs.**
**a,** Common binding motifs in 5' UTRs of E16 translationally de-repressed mRNAs identified members of Elav and Celf RNA binding protein family.
**b,** Common binding motifs in 5' UTRs of E16 translationally repressed mRNAs identified members of Elav and Celf RNA binding protein family.
**c,** Representative confocal images of E13 and E16 WT neocortex IHC for Celf1 (red) (n = 12 animals). DAPI shown in blue.
**d,** Representative confocal images of E13 VZ of WT (n = 6 animals) and *Celf1* KO (n = 2 animals) IHC for Celf1 (red). DAPI shown in blue.

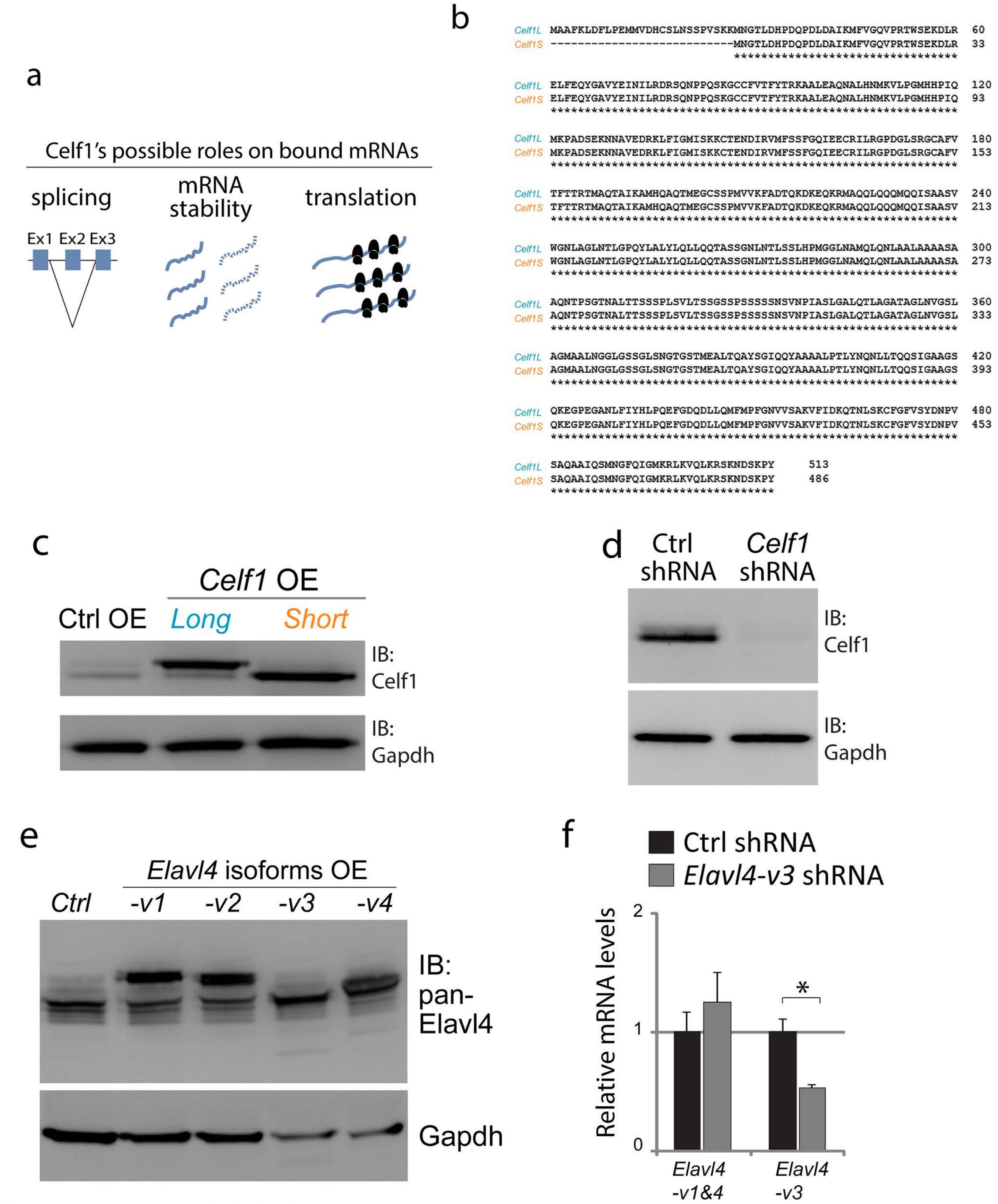


**Supplementary Figure 4. Validation of constructs.**

**a,** Possible roles of *Celf1* on bound mRNAs.

**b,** Alignment of Celf1L and Celf1S protein sequences.

**c,** N2a cells were transfected with either Control (Ctrl), *Celf1 long* (*Celf1L*) or *Cef1 short* (*Celf1S*) isoform-expressing constructs. Immunoblot for Celf1 (above) and the loading control Gapdh (below) (n = 6 transfections).

**d,** N2a cells were transfected with either Control or *Celf1* shRNA. Immunoblot for Celf1 (above) and the loading control Gapdh (below) (n = 6 transfections).

**e,** Western blot analysis of N2a cells transfected with each *Elavl4* isoform and control OE (leftmost lane).

**f,** qRT-PCR analysis of *Elavl4-v1&4* vs. *Elavl4-v3* in N2a cells transfected with an *Elavl4-v3* shRNA vector (n = 3 transfections). Data represent the mean and SD. Statistics: Student's t-test. *$p < 0.05$.

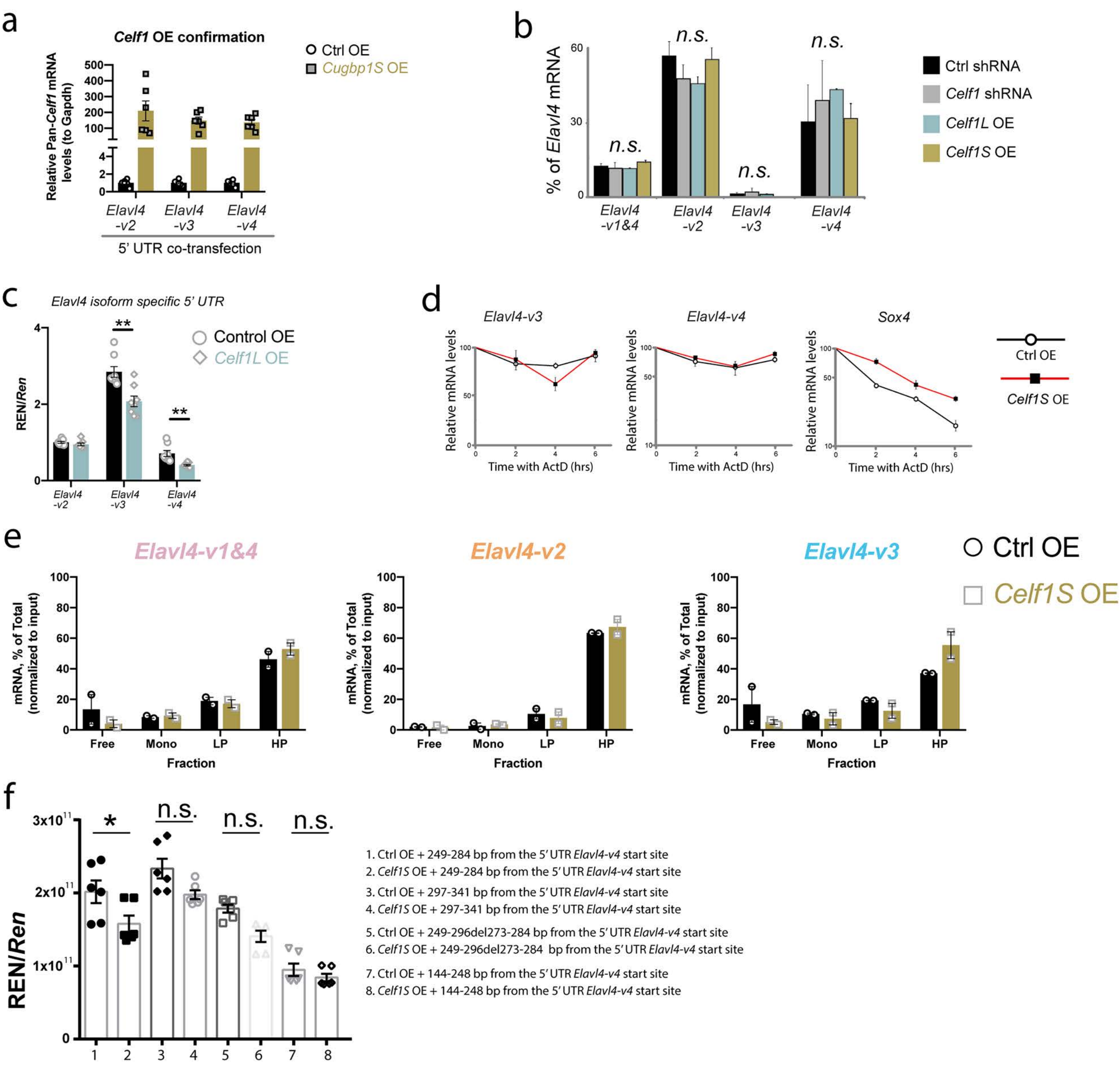


**Supplementary Figure 5. *Celf1S* OE does not alter splicing nor decay of *Elavl4* isoforms.**

**a,** Confirmation of *Celf1* overexpression 5' UTR of distinct *Elavl4* isoforms upstream of Renilla were co-transfected (n = 6 transfections). qRT-PCR for *Celf1* in Ctrl or *Celf1S* OE experimental condition. Statistics: Student's t-test. *** $p < 0.001$.

**b,** N2a cells were transfected with either Control (Ctrl) shRNA, *Celf1* shRNA, *Celf1L* OE, or *Celf1S* OE vectors (n = 6 transfections). *Elavl4* isoform expression was measured by qRT-PCR and normalized to *Gapdh*. Elavl4 isoform expression was detected under each condition. Data represent the mean and SEM. Statistics: one-way ANOVA with a Tukey test for multiple comparisons. n.s. = not significant.

**c,** N2a cells were transfected with Ctrl OE or *Celf1L* and plasmids with *Elavl4-v2*, *Elavl4-v3*, and *Elavl4-v4* specific 5' UTRs cloned upstream of *Renilla*. Translational output was quantified by measuring Renilla relative light units (REN) over *Renilla* mRNA levels (*Ren*) (n = 9 transfections). Data represent the mean and SEM. Statistics: Multiple t-tests. *** $p < 0.001$, n.s. = not significant.

**d,** N2a cells were transfected with Control or *Celf1S* OE vectors. 48 hours later, actinomycin-D (5 µg/mL) was added. *Elavl4-v3*, *Elavl4-v4*, and *Sox4* levels were measured at 0, 2, 4, and 6 hours after by qRT-PCR. Data are representative of 3 independent experiments with each in triplicates and presented as mean and SEM. *Celf1* OE modulates the stability of a known target mRNA *Sox4,* but not *Elavl4-v3* and *Elavl4-v4* (not significant by the Extra sum-of-squares F test).

**e,** polysome fractionation of N2a cells transfected with either Ctrl OE or *Celf1* OE. % of each isoform within free, monosome (mono), light polysome (light P), and heavy polysome (heavy P) fractions is shown. n = 2 fractionations, 1 transfection 10 cm plate per experiment.

**f,** Deletion strategy to determine Celf1-regulatory regions in 5' UTRs of *Elavl4-v1&4* (**Figure 4f**).

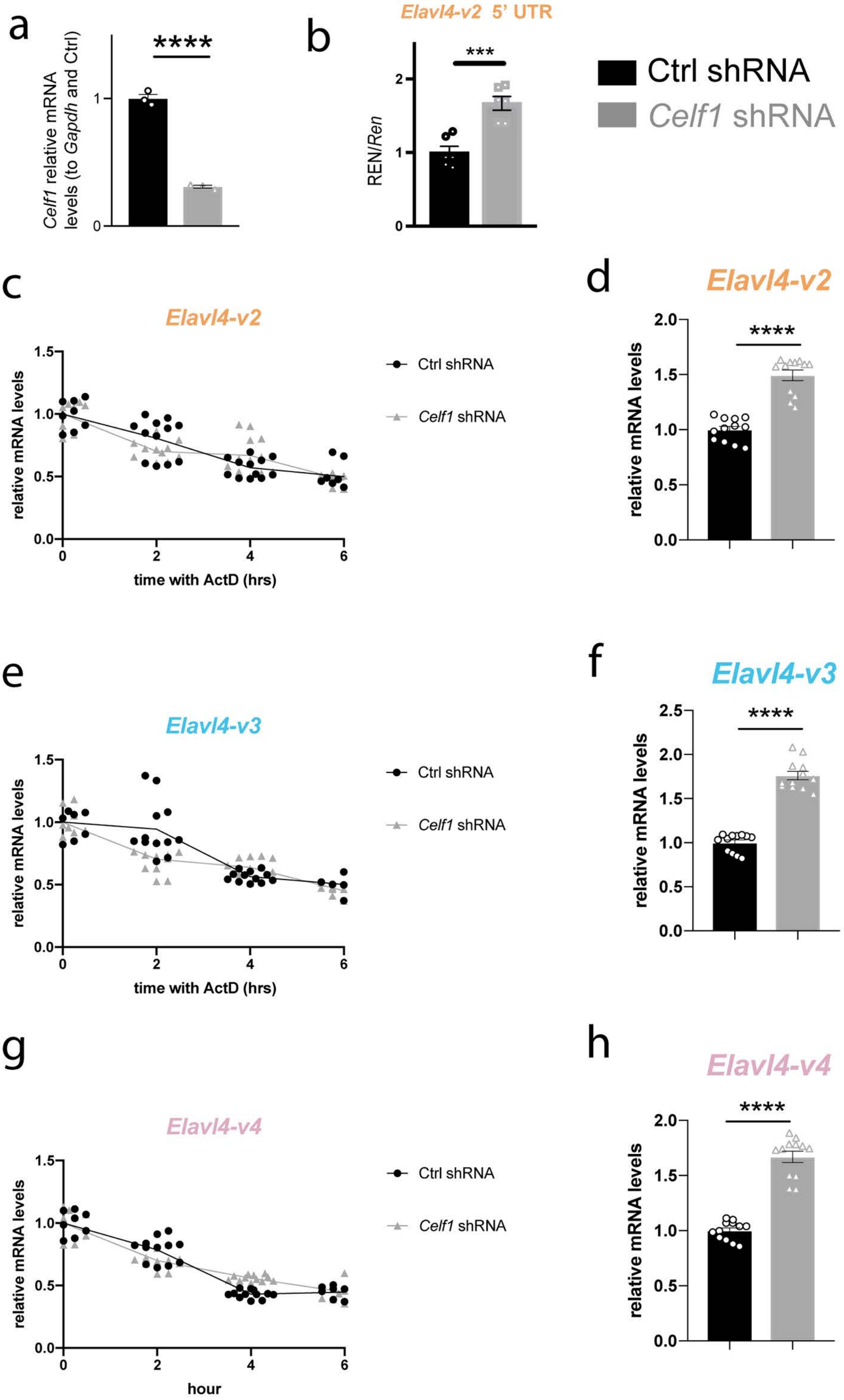


**Supplementary Figure 6. *Celf1* knockdown increases steady state mRNA levels of *Elavl4* isoforms but does not affect their decay.**

**a,** N2a cells were transfected with either Control (Ctrl) or *Celf1* shRNA. 72 hours later, cells were harvested for qRT-PCR analysis of mRNA levels (n = 6 transfections). *Celf1* was normalized to *Gapdh*. Statistics: Student's t-test. *** $p < 0.001$.

**b,** N2a cells were transfected with either Control (Ctrl) or *Celf1* shRNA and *Elavl4-v2* 5' UTR Renilla. Translational output was quantified by measuring Renilla relative light units (REN) over *Renilla* mRNA levels (*Ren*) (n = 9 transfections). Data represent the mean and SEM. Statistics: Student's T-test. * $p < 0.05$.

**c, e, g,** N2a cells were transfected with Control or *Celf1* shRNA vectors. 72 hours later, fresh media with actinomycin-D (5 µg/mL) was added to the cells to block transcription. *Elavl4* isoform levels were measured at 0, 2, 4, and 6 hours after the administration of actinomycin-D by qRT-PCR for mRNA decay (n = 6 transfections and treatments). Data represent the mean and SEM. No statistical significance was found for any isoform by the Extra sum-of-squares F test.

**d, f, h,** N2a cells transfected with Control or *Celf1* shRNA vectors for 72 hours (n = 6 transfections). qRT-PCR analysis of distinct *Elavl4* isoforms was normalized to *Gapdh* and Ctrl shRNA. Data represent the mean and SEM. Statistics: Student's t-test. *** $p < 0.001$.

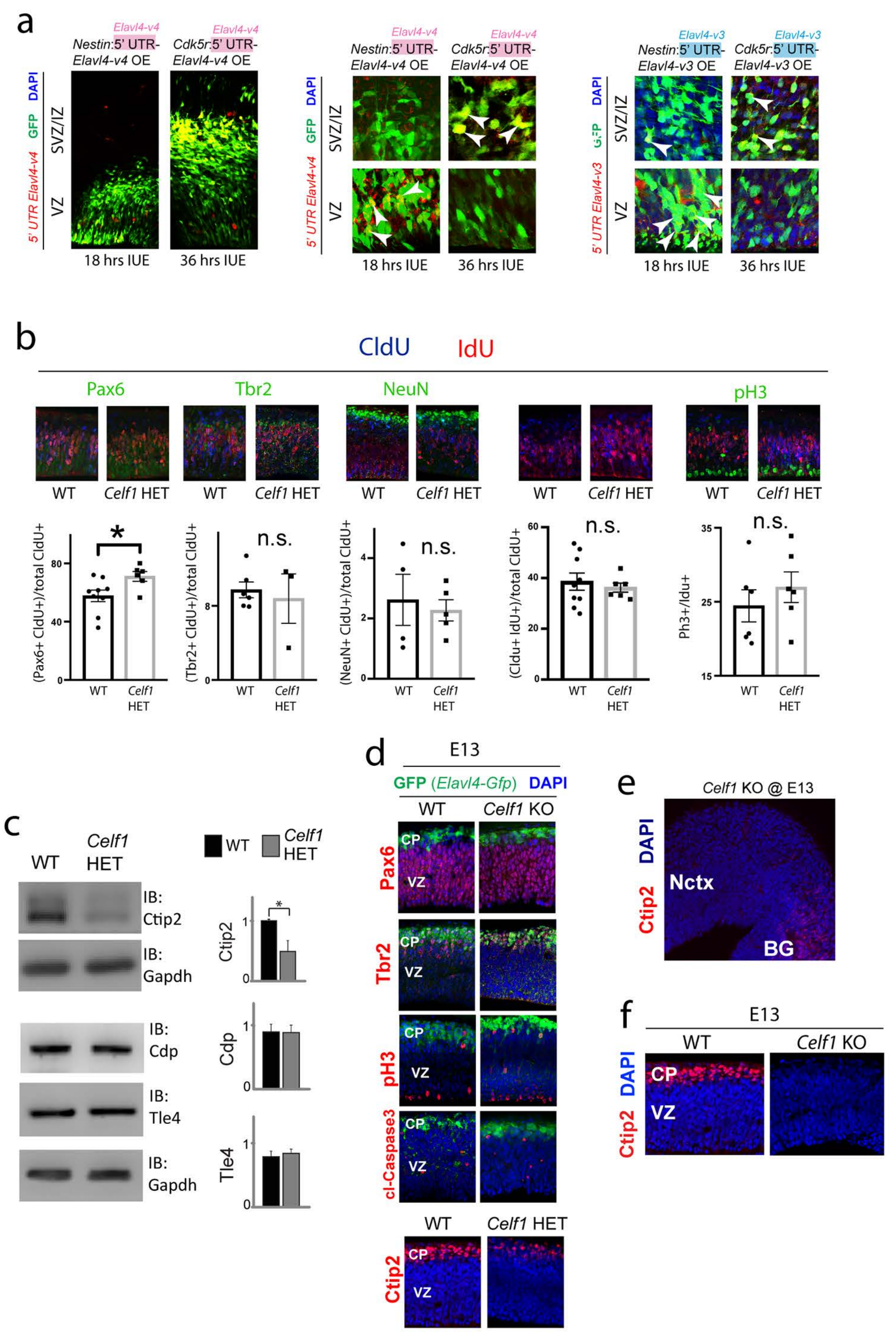


**Supplementary Figure 7. *Celf1* decrease does not alter all neocortical cell identity markers *in vivo*.**

**a,** Representative confocal images of E14 and E15 neocortices transfected with constructs overexpressing *5'UTR-Elavl4-v3* or *5'UTR-Elavl4-v4* under either *Nestin* or *Cdk5r* promoters. Cdk5r images were taken at 36 hours to allow for a cycle of mitosis. Confirmation of expression in appropriate transfected cell (green) using FISH for specific 5' UTRs (red, arrowhead). DAPI shown in blue.

**b,** E13 WT and *Celf1* HET neocortices were analyzed for changes in neurogenesis. We did not find higher cell cycle re-entry (Cldu+ IdU+ over total CldU+), changes in S-phase over M-phase ratio (IdU/pH3), IP production (labeled with Tbr2), or neuron production (labeled with NeuN). However, we did find higher retention of RG Pax6+ progenitors (leftmost). Since Elavl4 protein is increased in *Celf1* HETs, this is consistent with the phenotype typical of increased Elavl4 expression and is in line with previous findings[28,29]. (N = 3 animals for each experimental condition) Data represent the mean and SEM. Statistics: Student's t-test. n.s. = not significant, * $p < 0.05$.

**c**, Western blots (left) of WT and *Celf1* HET E13 neocortices for Ctip2, Cdp and Tle4 identity markers (n = 6 blots, 1 animal per blot). Gapdh was used as loading control. Densitometry quantification of westerns is shown (right). Data represent the mean and SD. Statistics: Student's t-test. n.s. = not significant, * $p < 0.05$.

**d, f,** Representative confocal images of E13 WT and *Celf1* KO neocortices show *Elavl4*-GFP (green) expression and IHC for (in descending order, all red) (n = 2 animals each): the radial glia marker Pax6, the intermediate progenitor/immature neuron marker Tbr2, the proliferative marker pH3, the apoptotic marker cleaved caspase 3, the UL marker Cdp and the LL marker Ctip2. DAPI is shown in blue. VZ = ventricular zone.

**e** , Low magnification image of E13 *Celf1* KO frontal section containing basal ganglia (BG) and neocortex IHC for Ctip2 (red). DAPI is in blue.

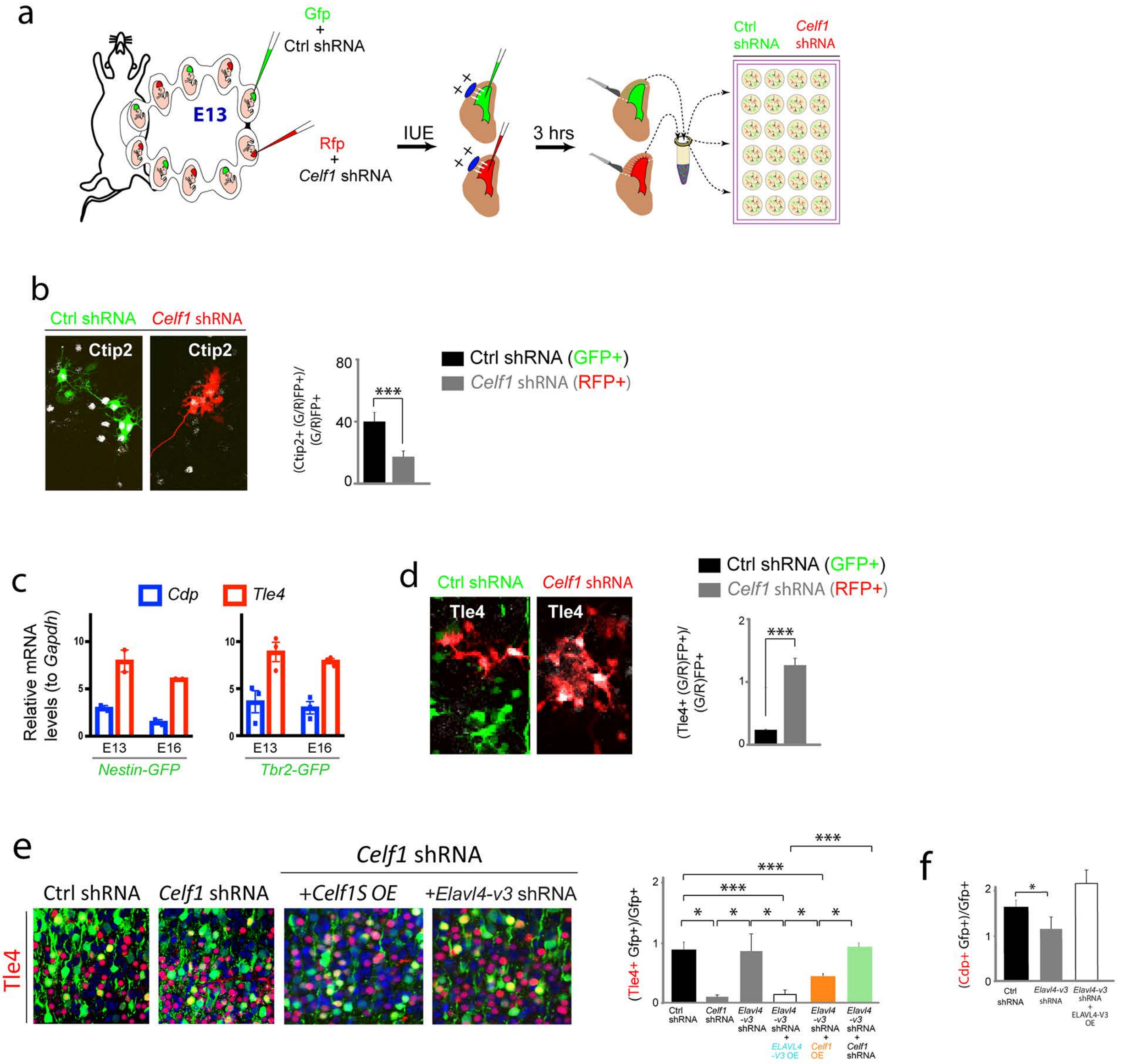


**Supplementary Figure 8. *Celf1* OE can induce subcortical projections from ULs.**

**a,** Schematic: GFP+*Ctrl* shRNA and RFP+*Celf1* shRNA were IUE in alternating E13 embryos (left). After 3 hours *in vivo*, primary neuronal cocultures were plated and cultured DIV3 (right), and IHC for layer markers.

**b,** Representative images of 3 DIV primary neocortical cocultures (n = 3 IUEs). IHC for RFP (red), GFP (green), and Ctip2 (white). (right) Quantification of Ctip2 colocalization. Data represent the mean of neurons and SEM. Statistics: Students t-test. *** $p < 0.001$.

**c**, Relative mRNA levels determined by qRT-PCR for UL identity marker *Cdp* and LL identity marker *Tle4* (n = 3 FACs experiments for all strains and ages, except Nestin-E16 n = 2) from FACS sorted GFP+ cells. Data represent the mean and SEM. Statistics: Student's t-test, 2-tailed, paired.. *** $P < 0.001$.

**d,** Representative images of 3 DIV primary neocortical co-cultures (n = 3 IUEs). IHC for RFP (red), GFP (green), and Tle4 (white). (right) Quantification of Tle4 colocalization. Data represent the mean and SEM. Statistics: Student's t-test. *** $p < 0.001$.

**e,** Representative confocal images at P0 of neocortices that underwent IUE at E13 and IHC at P0 for GFP (IUE cells, green) and LL identity marker Tle4 (red). (right) Quantification of IUEs for Tle4 layer identity marker. Data represent the mean of sections and SEM. Statistics: one-way ANOVA with a Tukey test for multiple comparisons. n = 3-5 per experimental condition. * $p < 0.05$, *** $p < 0.001$.

**f,** Quantification of IUEs for Cdp layer identity marker. Data represent the mean of sections and SEM. Statistics: one-way ANOVA with a Tukey test for multiple comparisons. n = 3-4 per experimental condition. * $p < 0.05$.

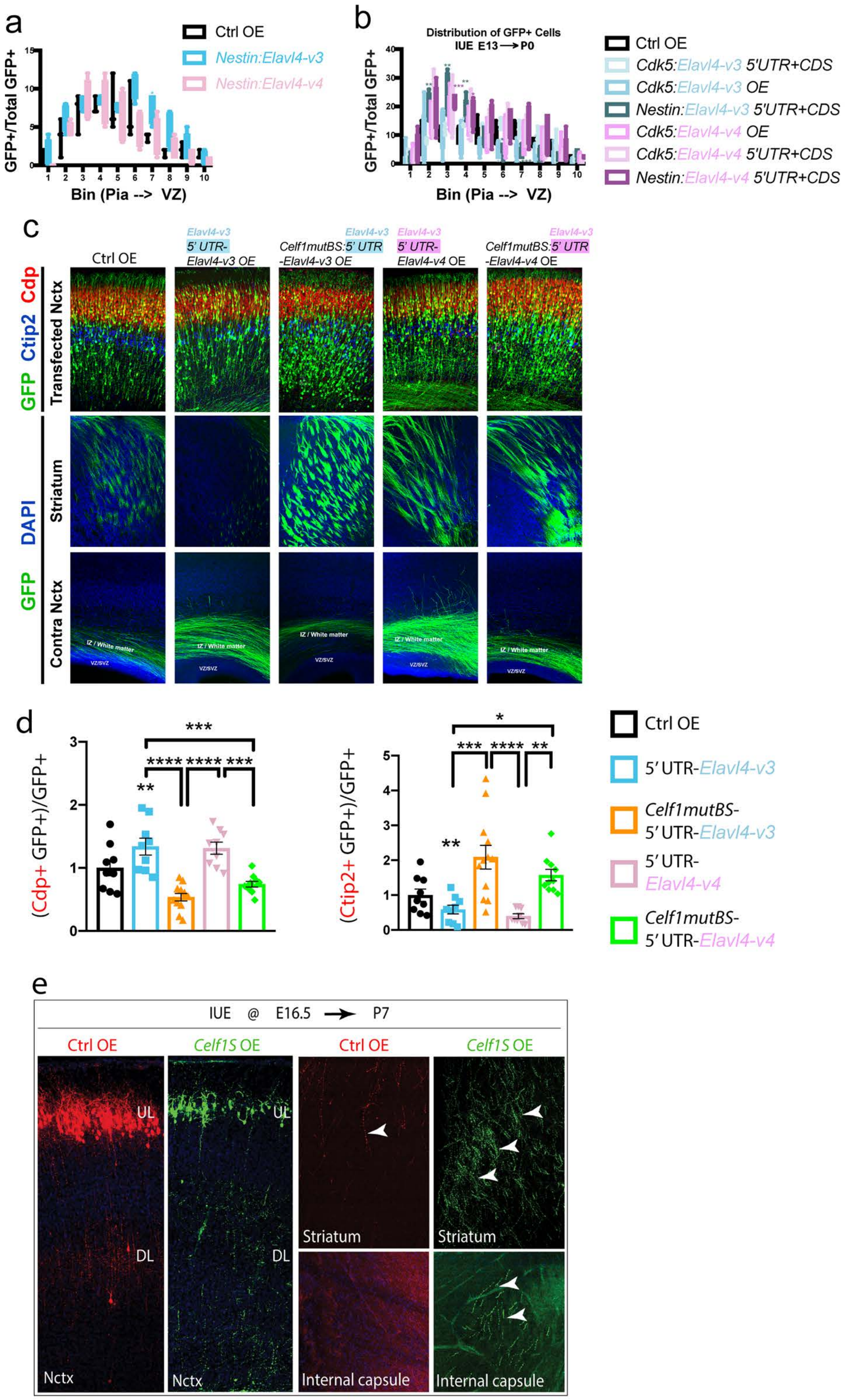


**Supplementary Figure 9.** ***Celf1*** **OE can induce subcortical projections from ULs, while mutation of Celf1 binding sites (BS) in 5' UTRs of** ***Elavl4-v3*** **or** ***Elavl4-v4*** **changes pathologies induced by native 5' UTR.**

**a, b,** Quantification of GFP+ cells in transfected cortices presented in main Fig. 6 **(a)** and main Fig. 7 (**b**) that were split into equal 10 bins from pial surface marginal zone (bin 1) to ventricular zone (bin 10) for the relative distribution quantifications. Two-way ANOVA with Tukey's. n.s. = not significant. *: $p < 0.05$; **: $p < 0.01$; ***: $p < 0.001$.

**c,** P0 representative confocal images of E13 IUE neocortices electroporated with GFP and either Ctrl (n = 3 animals), *5'-UTR-Elavl4-v3* (n = 3 animals), Celf1 binding site mutated 5' UTR of *Elavl4-v3* (*Celf1BSmut 5' UTR-Elavl4-v3)* (n = 3 animals), *5'-UTR-Elavl4-v4* (n = 3 animals) and *Celf1BSmut 5' UTR-Elavl4-v4* (n = 2 animals) OE plasmids. IHC for GFP (green), and cortical layer identity markers Cdp (red) and Ctip2 (blue).

**d ,** Quantification of **c**. Data represent the mean and SEM. Statistics: One-way ANOVA. *: $p < 0.05$; **: $p < 0.01$; ***: $p < 0.001$; ****: $p < 0.0001$.

**e,** Neocortices underwent IUE at E16.5 when intracortically-projecting neurons are being born. One hemisphere of the same brain underwent IUE with Ctrl OE and RFP (left panel), while the opposite hemisphere underwent IUE with *Celf1S* OE and GFP (right panel). Brains were analyzed at P7. Representative confocal images of neocortices, striatum, and internal capsule are taken from the same brain. Subcortical projections are marked with arrowheads. DAPI is in blue. Nctx=neocortex, UL =upper layers, DL = lower layers.

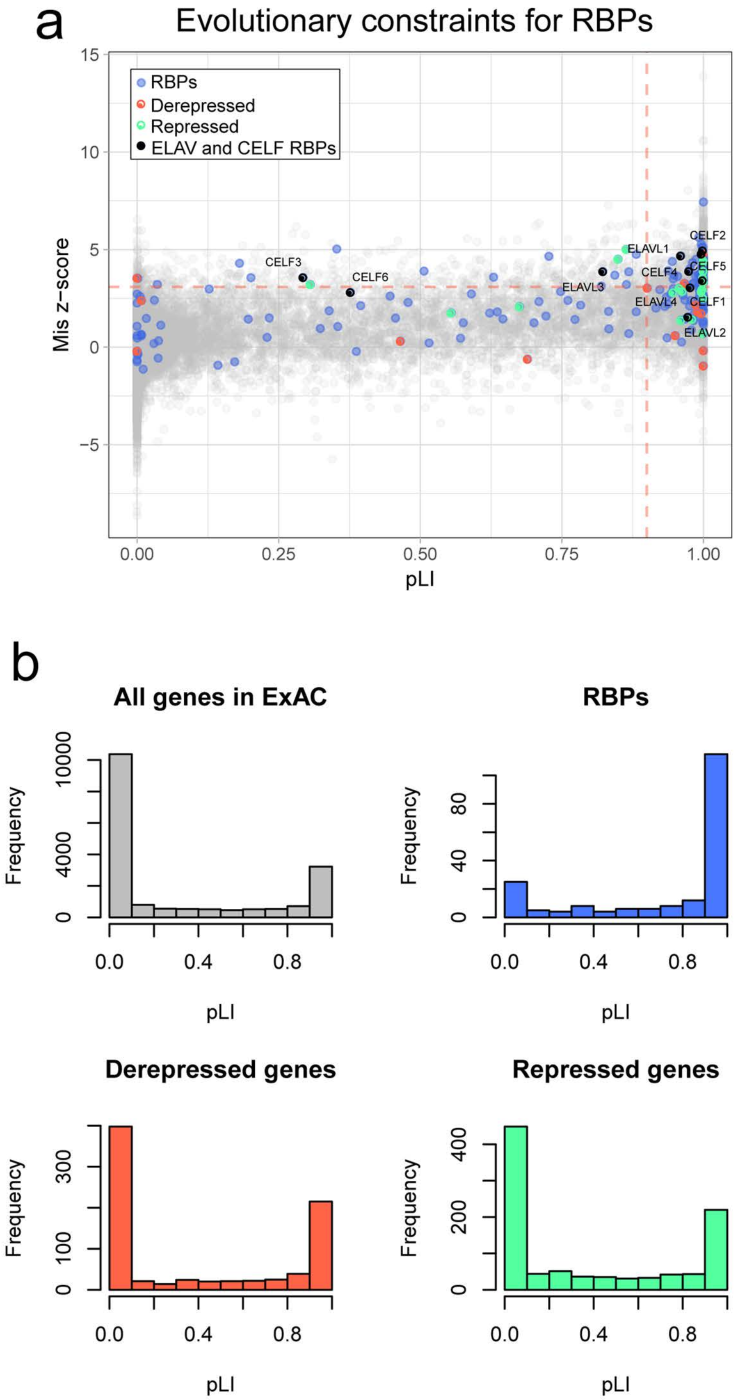


**Supplementary Figure 10. Intolerance to loss-of-function variants in genes encoding RNA-binding proteins and human orthologs of de-repressed and repressed genes at E16.**
**a,** Evolutionary constraints for RBPs and genes translationally repressed and derepressed at E16. The scatter plot shows the distribution of genes based on their probability of intolerance (pLI, y-axis) and missense z-score (x-axis) computed by the Exome Aggregation Consortium (ExAC) for all genes covered in ExAC (grey), RBPs (blue), human orthologs of the murine mRNAs translationally de-repressed at E16 (red), and human orthologs of the murine mRNAs translationally repressed at E16 (green). Red dotted lines represent thresholds for significant constraints. ELAVL and CELF family proteins are indicated in black. Data for this figure were used from the publicly available Exome Aggregation Consortium (ExAC), Cambridge, MA (URL: http://exac.broadinstitute.org)
**b,** The histograms show the frequency distribution of all genes in ExAC (grey), RNA-binding proteins (RBPs; blue), human orthologs of the murine mRNAs translationally derepressed at E16 (red), and human orthologs of the murine mRNAs translationally repressed at E16 (green) according to their pLI. Frequency distributions of RBPs and human orthologs of de-repressed and repressed genes differ from the reference distribution of all genes covered by ExAC (Two-sample Kolmogorov-Smirnov test, p-value<2.2e-16 for RBPs, p-value=2.41E-08 for derepressed genes, and p-value=4.09E-13 for repressed genes) and demonstrate intolerance to LOF variants.

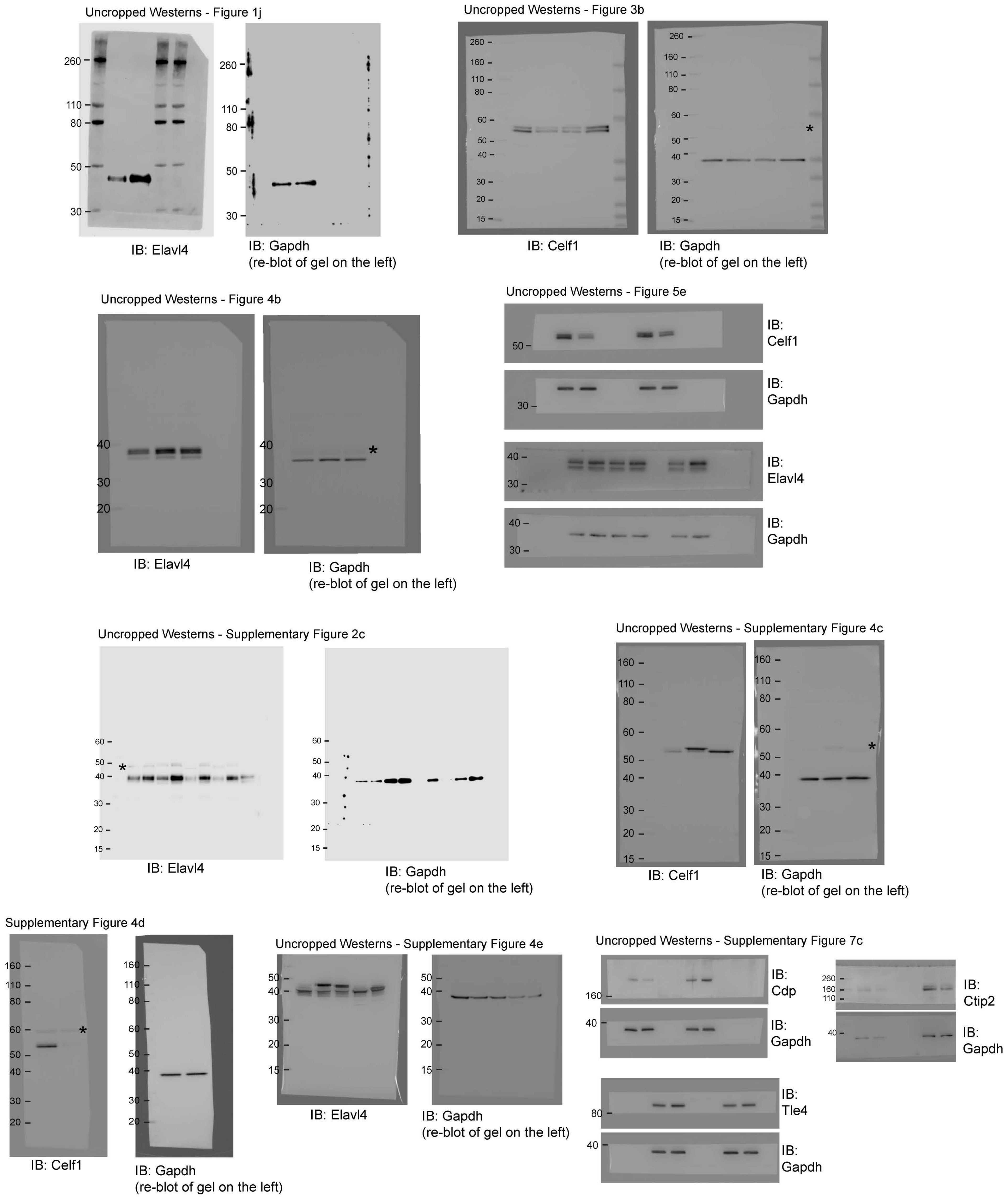


**Supplementary Figure 11. Uncropped Western blots used in this manuscript.** Corresponding figure is labeled on the top with ladders on the side and immunoblotting (IB) antibody.
*, left overs after blot stripping.
