# Supplementary material for: Translational derepression of Elavl4 isoforms at their alternative 5′ UTRs determines neuronal development
Source: Nat Commun. 2020 Apr 3;11:1674. doi: 10.1038/s41467-020-15412-8 (PMC7125149; doi:10.1038/s41467-020-15412-8)
Supplement: Supplementary file 2 — Description of Additional Supplementary Information [file 41467_2020_15412_MOESM2_ESM.pdf]

Description of Additional Supplementary Files

File Name: Supplementary Data 1
Description: Derepressed and repressed mRNAs at E16.

File Name: Supplementary Data 2
Description: Derepressed and repressed mRNAs encode risk genes associated with neurodevelopmental disorders.

File Name: Supplementary Data 3
Description Derepressed and repressed mRNAs are known FMRP targets.

File Name: Supplementary Data 4
Description: Additional primers list.
